# Contribution of NMDA Receptors to Synaptic Function in Rat Hippocampal Interneurons

**DOI:** 10.1523/ENEURO.0552-20.2021

**Published:** 2021-08-10

**Authors:** Sam A. Booker, Anna Sumera, Peter C. Kind, David J. A. Wyllie

**Affiliations:** 1 Centre for Discovery Brain Sciences, University of Edinburgh, Edinburgh EH8 9XD, United Kingdom; 2 Simons Initiative for the Developing Brain, University of Edinburgh, Edinburgh EH8 9XD, United Kingdom; 3 Patrick Wild Centre, University of Edinburgh, Edinburgh EH8 9XD, United Kingdom; 4 Centre for Brain Development and Repair, Institute for Stem Cell Biology and Regenerative Medicine, Bangalore 560065, India

**Keywords:** hippocampus, interneuron, long-term potentiation, NMDA receptor, receptor subunits, whole-cell patch-clamp

## Abstract

The ability of neurons to produce behaviorally relevant activity in the absence of pathology relies on the fine balance of synaptic inhibition to excitation. In the hippocampal CA1 microcircuit, this balance is maintained by a diverse population of inhibitory interneurons that receive largely similar glutamatergic afferents as their target pyramidal cells, with EPSCs generated by both AMPA receptors (AMPARs) and NMDA receptors (NMDARs). In this study, we take advantage of a recently generated GluN2A-null rat model to assess the contribution of GluN2A subunits to glutamatergic synaptic currents in three subclasses of interneuron found in the CA1 region of the hippocampus. For both parvalbumin-positive and somatostatin-positive interneurons, the GluN2A subunit is expressed at glutamatergic synapses and contributes to the EPSC. In contrast, in cholecystokinin (CCK)-positive interneurons, the contribution of GluN2A to the EPSC is negligible. Furthermore, synaptic potentiation at glutamatergic synapses on CCK-positive interneurons does not require the activation of GluN2A-containing NMDARs but does rely on the activation of NMDARs containing GluN2B and GluN2D subunits.

## Significance Statement

NMDA receptors (NMDARs) are ionotropic glutamate receptors that play a critical role in interneuronal communication, and learning and memory. Despite much being known about NMDARs and the role different subunits play in controlling principal cell activity, less is known about their function in inhibitory interneurons. Here, we use a recently developed rat line where the key GluN2A receptor subunit is removed, combined with subunit-specific pharmacology determine the synaptic properties and role of NMDAR subunits in interneuron function. Notably, we show that cholecystokinin-containing interneurons lack synaptic GluN2A and that long-term potentiation at glutamatergic synapses on them is mediated by GluN2D subunits. Our findings have ramifications for the etiology of neuropathological states and basic properties of brain function.

## Introduction

Neuronal networks require finely balanced excitatory glutamatergic and inhibitory GABAergic synaptic transmission to allow learning, memory, and typical brain function. Alterations to this balance may result in autism and intellectual disability (Antoine et al., 2019), epilepsy (Sloviter, 1987), schizophrenia (Daskalakis et al., 2002), and depression (Czéh et al., 2015). This balance has been well described in the CA1 of the hippocampus, where local inhibition arises from a heterogeneous population of inhibitory interneurons (INs) forming dense connections with themselves and excitatory pyramidal cells (PyrCs; Pelkey et al., 2017; Booker and Vida, 2018). INs possess diverse dendritic arbors, receiving glutamatergic inputs from both local and distant sources where synaptically released glutamate binds to and activates ligand-gated ion channels: chiefly AMPAreceptors (AMPARs) and NMDA receptors (NMDARs), that give rise to short (<10 ms) and long (100–1000 ms) synaptic events respectively (Cull-Candy et al., 2001). NMDARs have been proposed as the archetypal receptor underlying synaptic plasticity, and thus memory formation, in many cell types (Morris et al., 1990; Tsien et al., 1996; Morris, 2013).

NMDARs exist as tetrameric receptor complexes comprised of two GluN1 and two GluN2 subunits, the latter existing as four separate gene products (GluN2A-D; Gielen et al., 2009; Wyllie et al., 2013). During early brain development, GluN2B and GluN2D are the dominant isoforms in the hippocampus (Monyer et al., 1994; Flint et al., 1997; Martel et al., 2009; McKay et al., 2012). In mature principal hippocampal neurons GluN2A and GluN2B subunits predominate while GluN2D subunits have been reported in some IN populations (Engelhardt et al., 2015; Perszyk et al., 2016; Garst-Orozco et al., 2020). Native NMDARs can exist as either diheteromers (i.e., GluN1/GluN2A) or as triheteromeric assemblies (i.e., GluN1/2A/2B) with ligand binding, receptor kinetics and ion channel conductance being conferred by the identity of the nature of the GluN2 subunits present (Stern et al., 1992; Wyllie et al., 1996; Gielen et al., 2009; Hansen et al., 2014). Indeed, the presence of GluN2A subunits confers fast kinetics, whereas the presence of GluN2B and GluN2D subunits confer increasingly slow kinetics (Monyer et al., 1994; Vicini et al., 1998; Wyllie et al., 1998; for review, see Wyllie et al., 2013). Synaptically released glutamate, together with glycine (or D-serine), binds to synaptic and extrasynaptic NMDARs, and at depolarized membrane potentials the relief of voltage-dependent Mg^2+^ block allows ion conduction (reviewed in Hansen et al., (2018)). This requirement of postsynaptic depolarization and presynaptic neurotransmitter release for NMDAR opening defines them as a coincidence detector, critical for the establishment of Hebbian plasticity (Seeburg et al., 1995). Given these properties, the stoichiometry of NMDARs subunits confer distinct functional synaptic properties (Stern et al., 1992; Cull-Candy et al., 2001; Wyllie et al., 2013). Several studies have aimed to identify the contribution of NMDAR subunits to the different excitatory and inhibitory neuronal populations. The current understanding is that mature CA1 PyrCs exclusively express GluN2A/B-containing NMDARs (Flint et al., 1997; Gray et al., 2011). Meanwhile, hippocampal INs possess varied AMPAR- and NMDAR-mediated responses dependent on cell type (Akgül and McBain, 2016), and all major IN classes appear to express RNA for GluN2A/B/D subunits (Perszyk et al., 2016). Functional NMDARs have been shown on a variety of IN subtypes, including those expressing parvalbumin (PV; Korotkova et al., 2010; Billingslea et al., 2014) and cholecystokinin (CCK; Kotzadimitriou et al., 2018), morphologically defined basket cells (BCs) and dendritic inhibitory cells (Matta et al., 2013), *oriens/alveus* INs (Hájos et al., 2002), and neurogliaform neurons (Chittajallu et al., 2017). Despite this, many studies have been performed in juvenile rodent models and have not been able to determine the relative role of GluN2A in IN subtypes, because of the paucity of pharmacological modulators of this receptor subunit. Moreover, GluN2A-containing NMDARs are proposed to contribute to the induction of long-term potentiation (LTP) and GluN2B-containing NMDARs contribute to both long-term depression and LTP (Liu et al., 2004; Volianskis et al., 2013), while GluN2D NMDARs may contribute to both short- and long-term plasticity (Volianskis et al., 2013; Tozzi et al., 2016). Thus, determining the functional properties of NMDARs in INs will provide insight into their synaptic recruitment and plasticity (Booker and Wyllie, 2021).

This study assesses the relative NMDAR-mediated synaptic currents and the contribution of GluN2 subunits in morphologically and immunohistochemically identified hippocampal INs from wild-type and GluN2A-null outbred rats. We achieve this by performing whole-cell patch-clamp recording from INs and PyrCs to examine the NMDAR-mediated synaptic current in the presence of pharmacological manipulation and the role of GluN2 subunits in the generation of synaptic plasticity.

## Materials and Methods

### Animals

All procedures were performed according to UK and University of Edinburgh guidelines and under the authority of Home Office Licence (P1351480E). GluN2A-null rats were generated through CRISPR/Cas9 deletion of the gene, with GluN2A protein loss shown previously (Strehlow et al., 2019). All rats were maintained on an outbred Long–Evans Hooded background, and were housed on a 12 h light/dark cycle with *ad libitum* access to food and water. Male rats were taken for recordings at 4–6 weeks of age to avoid potential confounds because of onset of the estrus cycle. All recordings were performed at 4–6 weeks of age. The average age of wild-type rats was 31.1 ± 0.5 d (*N* = 38 rats total), and 32.4 ± 0.8 d (*N* = 27 rats total) for GluN2A-null rats.

### Acute slice preparation

Acute brain slices were prepared as previously described (Booker et al., 2017). Rats were anesthetized with isoflurane and decapitated, and their brains were rapidly removed and placed in ice-cold carbogenated (95% O_2_/5% CO_2_) sucrose-modified artificial CSF (sucrose-ACSF; in mm): 87 NaCl, 2.5 KCl, 25 NaHCO_3_, 1.25 NaH_2_PO_4_, 25 glucose, 75 sucrose, 7 MgCl_2_, 0.5 CaCl_2_. Horizontal slices (400 μm) containing the hippocampus were cut on an oscillating-blade vibratome (model VT1200S, Leica). Slices were placed in a submerged holding chamber in sucrose-ACSF for 30 min at 35°C, then stored at room temperature.

### Whole-cell patch-clamp recordings

For electrophysiological recordings, slices were transferred to a submerged recording chamber perfused with prewarmed carbogenated ACSF (in mm: 125 NaCl, 2.5 KCl, 25 NaHCO_3_, 1.25 NaH_2_PO_4_, 25 glucose, 1 MgCl_2_, and 2 CaCl_2_, at a flow rate of 4–6 ml/min at 30 ± 1°C). Slices were visualized under infrared differential inference contrast microscopy with a digital camera (Orca 2, Hamamatsu; or SciCamPro, Scientifica) mounted on an upright microscope (model BX61-WI, Olympus; or Slicescope, Scientifica) with a 40× water-immersion objective lens [1.0 numerical aperture (NA), Olympus]. Whole-cell patch-clamp recordings were performed with an amplifier (Multiclamp 700B, Molecular Devices). Recording pipettes were pulled from borosilicate glass capillaries (outer diameter, 1.7 mm; inner diameter, 1 mm; Harvard Apparatus) on a horizontal electrode puller (model P-97, Sutter Instruments). For recordings, pipettes were filled with a either a Cs-gluconate-based (in mm: 140 Cs-gluconate, 4 CsCl, 0.2 EGTA, 10 HEPES, 2 MgATP, 2 Na_2_ATP, 0.3 Na_2_GTP, 10 Na_2_phosphocreatine, 2.7 biocytin, and 5 QX-314; pH 7.4, 290–310 mOsm) or a K-gluconate-based (in mm: 142 K-gluconate, 4 KCl, 0.5 EGTA, 10 HEPES, 2 MgCl_2_, 2 Na_2_ATP, 0.3 Na_2_GTP, 10 Na_2_phosphocreatine, and 2.7 biocytin; pH 7.4, 290–310 mOsm) internal solution, which gave 3–5 MΩ pipette tip resistances on filling. Cells were rejected if they required a holding current of more than −200 pA to maintain −70 mV voltage clamp, series resistance started at >30 MΩ, or series resistance changed by >25% over the course of the recording. The average change in series resistance was 11.6% [mean_(start)_ = 18.7 ± 5.8 (range, 7.0–29.9); mean_(end)_ = 18.4 ± 6.5 (range, 7.6–36.5); *p* = 0.45, paired Student’s *t* test].

Whole-cell recordings of pharmacologically isolated EPSCs were performed following wash-in of Cs-gluconate solution (∼2–5 min) to provide optimal voltage clamp, in the presence of picrotoxin (50 μm). EPSCs were generated by a twisted Ni:Chrome bipolar wire placed either in stratum radiatum (CA1 PyrC, PV-INs, CCK-INs) or in the alveus [somatostatin (SSt) INs], reflecting major synaptic inputs to different cell types (stimulus duration, 0.1 ms; stimulus, 20.3 ± 18.3 V). Stimuli were delivered via constant voltage stimulator (Digitimer) sufficient to produce a monosynaptic EPSC in the range of ∼200 pA. The amplitude of these monosynaptic AMPA-EPSCs varied between cell types (*p* = 0.0016, one-way ANOVA) but not within a cell type [CA1 PyrCs: *p *=* *0.84; PV INs: *p *=* *0.83; *p *=* *0.84; *p *=* *0.69; Holm–Sidak tests]. For AMPAR-EPSCs, 10 traces were collected at 20 s intervals at −70 mV. For NMDAR-EPSCs, either CNQX was bath applied first and AMPAR-EPSC blockade observed, or the cell was held at +40 mV to identify the mixed AMPAR/NMDAR-EPSCs, and then CNQX was applied. All NMDAR-EPSCs were recorded following full wash-in of CNQX at +40 mV using the same stimulation intensity as for AMPAR-EPSCs. Pharmacology for the specific NMDAR subunits was as follows: GluN2B-ifenprodil tartrate (10 μm) or NAB-14 (10 μm), and both were bath applied. As NAB-14 is highly lipophilic (Yi et al., 2019), all tubing was rinsed with 100% ethanol and liberal amounts of distilled water between recordings to ensure full removal of the drug from the surfaces of perfusion tubes. All EPSC amplitudes were recorded at the peak of the EPSC, as measured over a 2 ms peak average. All rise times are reported as the 20–80% of the peak EPSC amplitude. Decay time constants were calculated from a monoexponential (AMPAR-EPSC) or biexponential (NMDAR-EPSC) curve fit to the decaying phase of the EPSC. For biexponential fits, the weighted tau was taken as the decay time constant. For all kinetic properties, only NMDAR EPSCs with amplitude >20 pA were included for analysis, to exclude measurement artifacts. NMDAR/AMPAR EPSC amplitude ratios were calculated from the average EPSC recorded for the respective epoch.

For LTP recordings, cells were recorded in current clamp, using a K-gluconate-based internal solution. Putative CCK INs were selected for recording in *stratum radiatum* and monosynaptic EPSPs were generated via bipolar electrode stimulation of the *stratum radiatum*, placed ∼500 μm distal to the recorded somata. Following breakthrough into whole-cell configuration, the membrane potential was set to −70 mV with the application of a bias current, and the bridge was balanced. Then EPSPs with amplitude of ∼5 mV (mean, 5.94 ± 2.85 mV; range, 1.64–12.35 mV) were recorded for 5 min of stable baseline (<10% change over 5 min, calculated from a linear fit of baseline period); if stability was not achieved within 7.5 min of breakthrough, the recording was abandoned. Following 5 min of stable recording, LTP was induced, with 2 × 100 Hz of tetanus stimuli, at the same strength as the baseline EPSP. Following induction, 25 min of EPSPs were recorded to assess the potentiation of the whole-cell EPSP, which was reported as the peak amplitude measured as the peak 2 ms response. All recordings were filtered online at 10 kHz with the built-in four-pole Bessel filter and digitized at 20 kHz (Digidata1440, Molecular Devices). Traces were recorded in pCLAMP 9 (Molecular Devices) and stored on a personal computer. Analysis of electrophysiological data were performed offline using the open source software package Stimfit (Guzman et al., 2014).

Following recording, all cells were resealed by carefully withdrawing the patch-pipette in a manner akin to forming an outside-out patch. The slices were then fixed in 4% paraformaldehyde dissolved in 0.1 m phosphate buffer (PB) pH 7.35, overnight at 4°C. Slices were then transferred to 0.1 m PBS until processing for immunohistochemistry.

### Histologic processing and imaging

Immunolabelling of recorded neurons was performed as previously described (Booker et al., 2014), slices were washed several times in PBS, then blocked in 10% normal goat serum (NGS), 0.3% Triton X-100, and 0.05% NaN_3_ in PBS for 1 h at room temperature. Slices were then incubated with primary antibodies for PV (monoclonal mouse; 1:5000; SWANT), pre-pro-CCK (polyclonal rabbit; 1:1000; Frontiers Laboratory), or SSt (polyclonal rabbit; 1:2000; Peninsula Laboratories). Primary antibodies were diluted in PBS containing 5% NGS, 0.3% Triton X-100, and 0.05% NaN_3_ for 72 h at 4°C. Slices were thoroughly washed with PBS, and then secondary antibodies (anti-mouse or anti-rabbit; 1:1000; Alexa Fluor 488, Thermo Fisher Scientific) were applied with streptavidin conjugated to Alexa Fluor 633 (1:1000; Thermo Fisher Scientific) applied diluted in PBS containing 3% NGS, 0.1% Triton X-100 and 0.05% NaN_3_ for 3 h at room temperature or 24 h at 4°C. Slices were rinsed in PBS then PB, and were mounted on glass slides with VECTASHIELD HardSet Antifade Mounting Medium (catalog #H1400, Vector Laboratories). For CA1 PyrCs, the same protocol was used, albeit with streptavidin Alexa Fluor 633 applied at 1:1000.

Confocal image stacks were collected on an invert scanning-confocal microscope (LSM 510 META Confocal with Axiovert 200, Zeiss) equipped with a 20× (0.45 NA; Zeiss) air-immersion or a 63× (1.4 NA; Zeiss) oil-immersion objective lens. For the identification of neurons, *z*-stacks (1 μm steps; 1024 × 1024 pixels) containing the somatodendritic axis and axon distribution were carried out. For neurochemical identification, the somata were imaged under high magnification, and immunolabeling was assessed over the somatic focal plane. All image analysis was performed with the FIJI package of ImageJ. For reconstruction, the neurons were imaged fully then stitched, segmented, and rendered offline in FIJI, using the Simple Neurite Tracer (SNT) plug-in (Longair et al., 2011).

### Statistical analysis

All experiments and analysis were performed blind to genotype. Throughout this study, data are shown as the mean ± SD, and the number of cells (*n*) and animals (*N*) are indicated. The required sample size was estimated based on an assumed effect size of 20% with 15% SD at 80% power, giving a required *N* = 7–10/group. Some data showed a >20% effect size, thus requiring fewer biological replicates (*N*). All data are reported as animal averages, unless stated otherwise, and are shown alongside estimation statistics of difference between means and confidence intervals (CIs) in the form (*r* value, effect size, 95% confidence interval, *p* value, test performed), the usefulness of which has been demonstrated previously (Manouze et al., 2019). We performed statistical comparisons of effect size in a paired or unpaired manner, using Student’s *t* test, Mann–Whitney test, or Wilcoxon signed-rank test, depending on whether data were normally distributed, which was confirmed with the Anderson–Darling test. For group analysis, one-way ANOVA was performed. Statistical significance was assumed if *p* < 0.05.

### Data availability

The data that support the findings of this study are available from the corresponding author on reasonable request.

## Results

### Absence of GluN2A subunits leads to slowed kinetics of NMDAR-EPSCs in CA1 PyrCs

**Figure 1.**
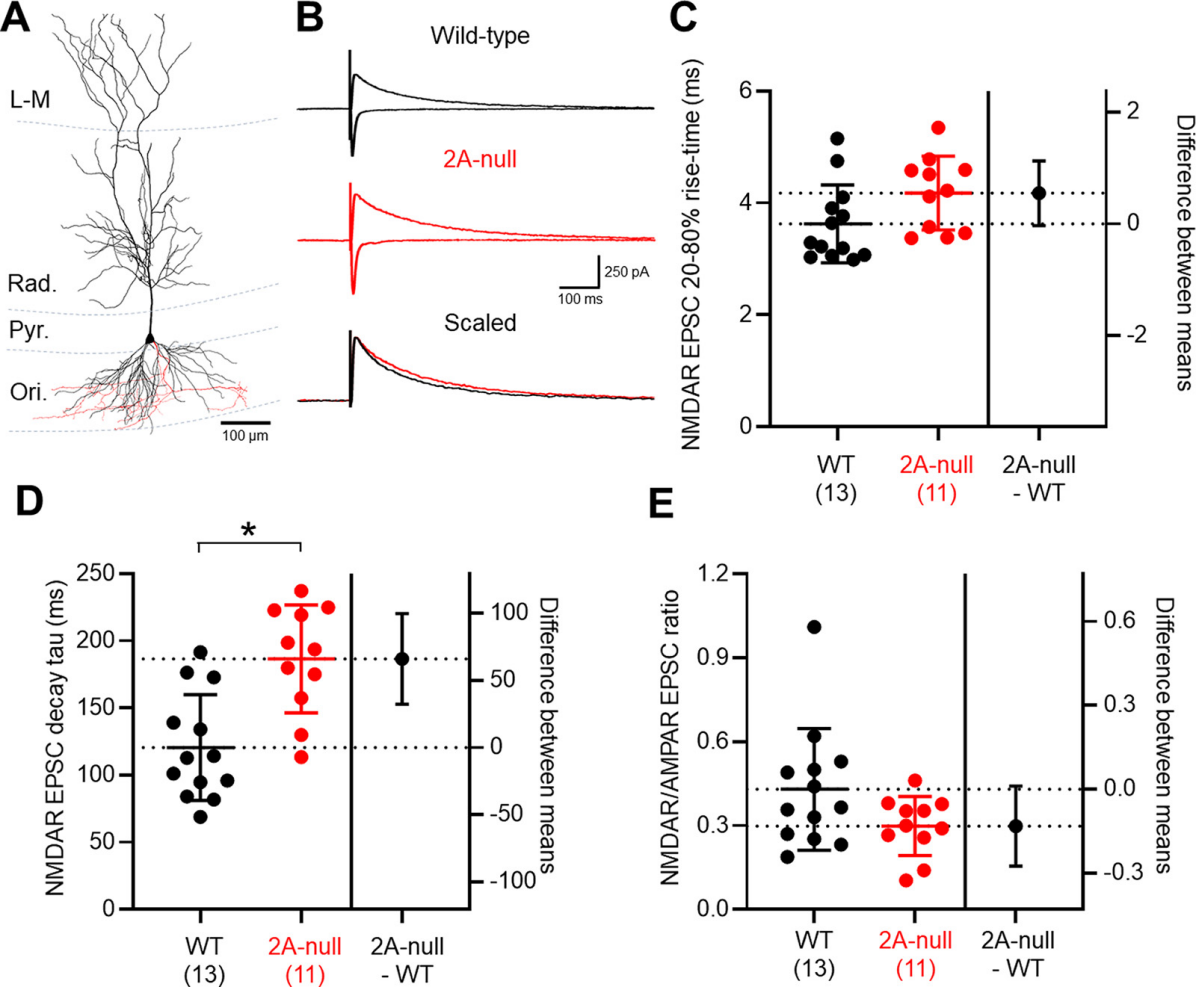
The absence of GluN2A confers slowed NMDAR kinetics on CA1 PyrCs. ***A***, Reconstruction of a CA1 PyrC showing orientation with respect to the hippocampal layers *stratum oriens* (Ori.), *pyramidale* (Pyr.), *radiatum* (Rad.), or *lacunosum-moleculare* (L–M). Somatodendritic axis (black) and axonal arborization (red) are indicated. ***B***, Representative EPSCs recorded from CA1 PyrCs from wild-type (black) and GluN2A-null (red) rats. AMPAR-EPSCs (inward currents) and NMDAR-EPSCs (outward currents) are shown. The peak scaled NMDAR traces are shown below to indicate the slowing of the NMDAR-EPSC. ***C***, NMDAR-EPSC rise times, quantified for all recorded CA1 PyrCs. Data from individual cells are shown as filled circles from wild-type rats (black; *n* = 20 cells; *N* = 13 rats) and GluN2A-null rats (2A-null, red; *n* = 21 cells; *N* = 11 rats). ***D***, NMDAR-EPSC decay time constants (tau) from the weighted tau of a biexponential curve fit. ***E***, NMDAR/AMPAR ratio of EPSCs elicited by Schaffer collateral stimulation. Fewer rats are shown for kinetic values because of the 20 pA cutoff imposed on kinetic data. Data are shown as the mean ± SD, alongside the difference between the means ± CI. *p < 0.05, Student’s *t* test.

To first confirm that loss of GluN2A receptors leads to functional changes in NMDAR signaling, we made recordings from identified CA1 PyrCs (Fig. 1*A*) in both wild-type (*n* = 20 cells; *N* = 13 rats) and GluN2A-null littermate rats (*n* = 21 cells; *N* = 11 rats). In all recordings, large AMPAR-mediated EPSCs were recorded in response to electrical stimulation of putative Schaffer collateral afferents in *stratum radiatum* (Fig. 1*B*). These EPSCs had an average amplitude of 357 ± 198 pA in wild-type rats, which was similar to that of 509 ± 228 pA in GluN2A-null rats (*r *=* *0.12, −152; CI, −332, +29; *p *=* *0.095, unpaired Student’s *t* test). The AMPAR rise time was 2.1 ± 0.7 ms and had a decay time constant of 8.5 ± 1.7 ms in wild-type rats, which was very similar in the GluN2A-null PyrCs (rise time: *r *=* *0.002, +0.053; CI, −0.46, 0.57; *p *=* *0.83; decay time constant: *r *=* *0.03, +0.80; CI, −1.23, 2.82; *p *=* *0.42, unpaired Student’s *t* tests). When recorded at +40 mV in the presence of 10 μm CNQX, we consistently observed NMDAR-EPSCs (Fig. 1*B*), which in wild-type rats had a rise time of 3.6 ± 0.7 ms and a weighted decay time constant of 120.5 ± 39.4 ms. The average NMDAR-EPSC had an amplitude of 129 ± 73 pA, giving rise to an average NMDAR/AMPAR ratio of 0.43 ± 0.22. Consistent with GluN2A subunits conferring rapid gating kinetics to NMDARs, we observed a slowing of NMDAR-EPSCs in the GluN2A-null rat (Fig. 1*B*) with 20–80% rise time slowed by 27% (*r *=* *0.15, +0.55; CI, −0.03, 1.12; *p *=* *0.06, unpaired Student’s *t* test; Fig. 1*C*) and the decay time constant slowed by 75% (*r *=* *0.13, +66.1; CI, 32.3, 99.9; *p *=* *0.0005, unpaired Student’s *t* test; Fig. 1*D*). We observed a 28% reduction in the NMDAR/AMPAR ratio associated with loss of the GluN2A subunit, which was not significantly different (*r *=* *0.43, −0.13; CI, −0.28, 0.02; *p *=* *0.08, unpaired Student’s *t* test; Fig. 1*E*), suggesting that the total number of NMDARs may be reduced at Schaffer collateral synapses. Together, these data confirm that NMDARs in CA1 PyrCs contain GluN2A subunits, which contribute to the kinetics properties of the receptor.

### NMDARs in hippocampal INs express variable levels of GluN2A

**Figure 2.**
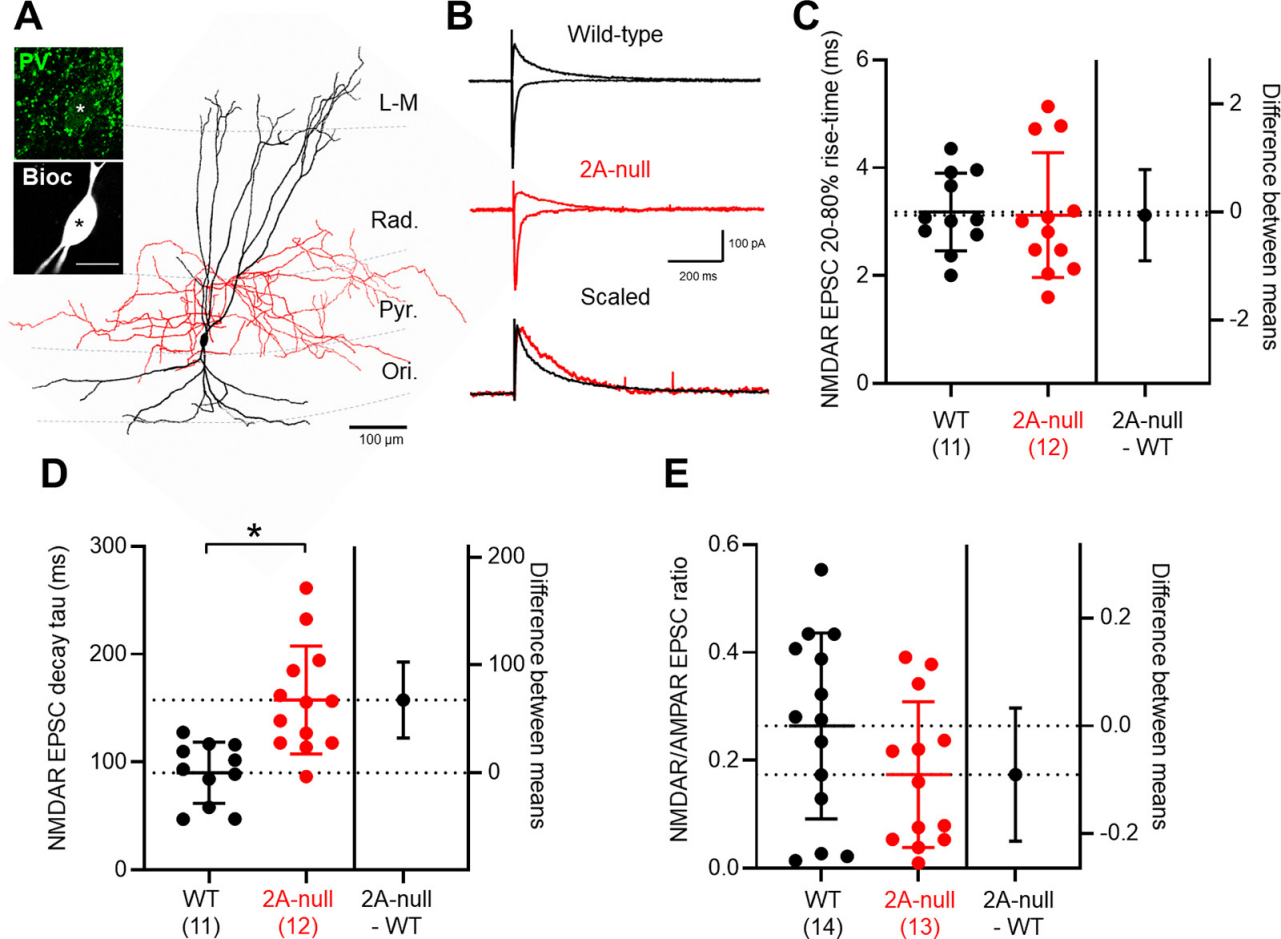
GluN2A subunits contribute to NMDAR-EPSCs in identified PV INs. ***A***, Reconstruction of a PV IN recorded in CA1 with respect to the hippocampal layers, with the somatodendritic axis indicated in black and axons localized to *stratum pyramidale* in red. Inset, Immunohistological labeling for PV (green) aligned (asterisk) to the biocytin-filled somata (black and white). Scale bar, 10 μm. ***B***, Representative monosynaptic AMPAR- and NMDAR-mediated EPSCs recorded from PV INs in wild-type (black) and 2A-null (red) rats. The scaled NMDAR-mediated EPSC (bottom) indicates the slowing of response in the GluN2A-null rat line. ***C***, NMDAR-EPSC rise times, quantified for identified PV-INs from wild-type (black; *N* = 14 rats) and 2A-null (red; *N* = 13 rats) rats, with individual cells shown overlain (filled circles); fewer rats are shown for kinetic values because of the 20 pA cutoff. ***D***, Quantification of NMDAR-mediated EPSC decay time constants (tau). ***E***, NMDAR/AMPAR ratio of EPSCs elicited by Schaffer collateral stimulation. Data are shown as the mean ± SD. *p < 0.05, Student’s *t* test.

We next assessed the contribution of GluN2A subunits to synaptic NMDARs in identified hippocampal INs. We first sought to identify EPSCs in PV INs, which were located in and around *stratum pyramidale*, with large somata with multipolar dendrites. These were initially selected based on low membrane resistance and fast membrane decay times on breakthrough into the whole-cell configuration. For this study we identified 20 PV INs from 15 wild-type rats, and 14 PV INs from 13 GluN2A-null rats, which all displayed clear immunolabelling for PV. In wild-type rats, 50% (*n* = 10 cells) of PV INs were identified as BCs with axons localized to *stratum pyramidale* (Fig. 2*A*) and 35% (*n* = 7 cells) had axons localized to *stratum radiatum* and *stratum oriens*, thus representing likely bistratified cells; the remaining three cells had axons cut close to the soma, thus preventing further identification. In GluN2A-null rats, 79% of recovered PV INs were BCs (*n* = 11 cells), 14% were bistratified (*n* = 2 cells), and one cell could not be identified because of a proximally cut axon.

In PV INs, the stimulation of *stratum radiatum* in the presence of picrotoxin (50 μm) at −70 mV gave rise to AMPAR-mediated EPSCs with amplitudes of 373 ± 306 pA in wild-type rats and 350 ± 166 pA in GluN2A-null rats (*r *=* *0.003, 24.1; CI, −173, 221; *p *=* *0.80, unpaired Student’s *t* test), with similar rise times (WT, 1.6 ± 1.6 ms; GluN2A-null, 1.6 ± 1.3 ms; *r *=* *0.0007, −0.08; CI, −0.08, 0.56; *p *=* *0.80, unpaired Student’s *t* test), but 33% longer decay time constants (WT, 6.7 ± 2.1 ms; GluN2A-null, 9.0 ± 3.4 ms; *r *=* *0.15, 2.3; CI, 0.06, 4.5; *p *=* *0.05, unpaired Student’s *t* test). NMDAR-mediated EPSCs, recorded at +40 mV in PV INs from WT rats (Fig. 2*B*) had an amplitude of 76 ± 62 pA, a 20–80% rise time of 3.2 ± 0.7 ms (Fig. 2*C*), and a decay time constant of 90.1 ± 28.4 ms (Fig. 2*D*), which was ∼30 ms faster than those of CA1 PyrCs (*r *=* *0.17, 30.4; CI, −60.0, −0.76; *p *= 0.045, unpaired Student’s *t* test). Because of the thresholding of NMDAR-EPSCs at 20 pA for kinetic measurements, three animals were excluded from these analyses. The NMDAR/AMPAR ratio for wild-type PV INs was 0.26 ± 0.17, which was lower than that of CA1 PyrCs (*r *=* *0.16, −0.17; CI, −0.32, −0.01; *p *=* *0.038, unpaired Student’s *t* test). In terms of cell type-specific effects, the decay time constants of PV BCs (109.2 ± 21.1 ms) tended to be longer than those of PV bistratified cells (78.0 ± 36.1 ms; *r *=* *0.25, −31.3; CI, −69.3, 6.8; *p *=* *0.097, unpaired Student’s *t* test), despite similar NMDAR/AMPAR ratios (*r *=* *0.007, 0.035; CI, −0.21, 0.28; *p *=* *0.77, unpaired Student’s *t* test). All kinetic data for identified subtypes are shown in Table 1.

**Table 1:** Key properties of NMDAR-mediated EPSCs in PyrCs and in morphotypes in CA1 of the hippocampus

Cell type	*N*		Decay time constant (ms)			NMDAR/AMPAR ratio		
	WT	2A null	WT	2A null	*p*	WT	2A null	*p*
PyrC								
All	13	7	120 ± 39	187 ± 40	**0.0007**	0.43 ± 0.22	0.30 ± 0.11	0.08
PV								
All	14	13	90 ± 28	158 ± 50	**0.0007**	0.26 ± 0.17	0.17 ± 0.14	0.14
BC	9	11	109 ± 21	156 ± 54	**0.0013**	0.27 ± 0.25	0.18 ± 0.20	0.40
Bistratified	6	2	78 ± 36	154 ± 51	n/a	0.30 ± 0.14	0.24 ± 0.22	n/a
SSt								
All	14	12	85 ± 28	159 ± 60	**0.0042**	0.46 ± 0.48	0.53 ± 0.51	0.90
OLM	9	8	76 ± 25	145 ± 27	**0.0005**	0.37 ± 0.38	0.43 ± 0.51	0.35
CCK								
All	27	16	170 ± 64	152 ± 44	0.34	0.63 ± 0.36	0.55 ± 0.36	0.53
BC	14	7	187 ± 51	133 ± 35	**0.0216**	0.50 ± 0.37	0.57 ± 0.44	0.69
SCA/ADA	15	5	193 ± 100	177 ± 38	0.86	0.61 ± 0.29	0.69 ± 0.18	0.46
PPA	5	2	160 ± 59	145 ± 10	n/a	0.81 ± 0.59	0.67 ± 0.17	n/a

Summary of NMDAR-mediated EPSC decay time constant and NMDAR/AMPAR ratio from identified morphotypes of hippocampal INs. Statistics are shown as *p* values reported from unpaired Student’s two-tailed *t* tests; where statistical significance was observed this has been indicated (bold). Data are shown as the mean ± SD.

Compared with wild-type rats, NMDAR-EPSCs recorded in PV INs from GluN2A-null rats had similar amplitudes of 51 ± 46 pA (*r *=* *0.54, −25.1; CI, −68.5, 18.3; *p *=* *0.25, unpaired Student’s *t* test) and rise times of 3.1 ± 1.2 ms (*r *=* *0.001, −0.06; CI, −0.91, 0.79; *p *=* *0.89, unpaired Student’s *t* test). Consistent with the contribution of GluN2A subunits to NMDAR-EPSCs evoked in PV INs, we measured a 75% increase in the decay time constant to 158 ± 50 ms in GluN2A-null rats compared with wild-type rats (*r *=* *0.42, 67.5; CI, 32.2, 102.8; *p *=* *0.0007, unpaired Student’s *t* test; Fig. 2*D*). PV INs in GluN2A-null rats had a NMDAR/AMPAR ratio of 0.17 ± 0.14, which was lower but not significantly different from that of WT rats (*r *=* *0.084, −0.09; CI, −0.21, 0.03; *p *=* *0.14, unpaired Student’s *t* test; Fig. 2*E*). Although not statistically different between genotypes, the decay kinetics for identified BCs and bistratified cells generally obeyed the population average (Table 1). Consistent with their decay time constants in wild-type rats, BCs showed an ∼50% slowing of the NMDAR-EPSC decay in GluN2A-null rats, while bistratified cells showed 200% slowing in decay time constants (Table 1). These data suggest that GluN2A-containing NMDARs contribute to synaptic currents in PV-INs, comparable to that of CA1 PyrCs, with noted differences between the morphologic subtypes identified.

**Figure 3.**
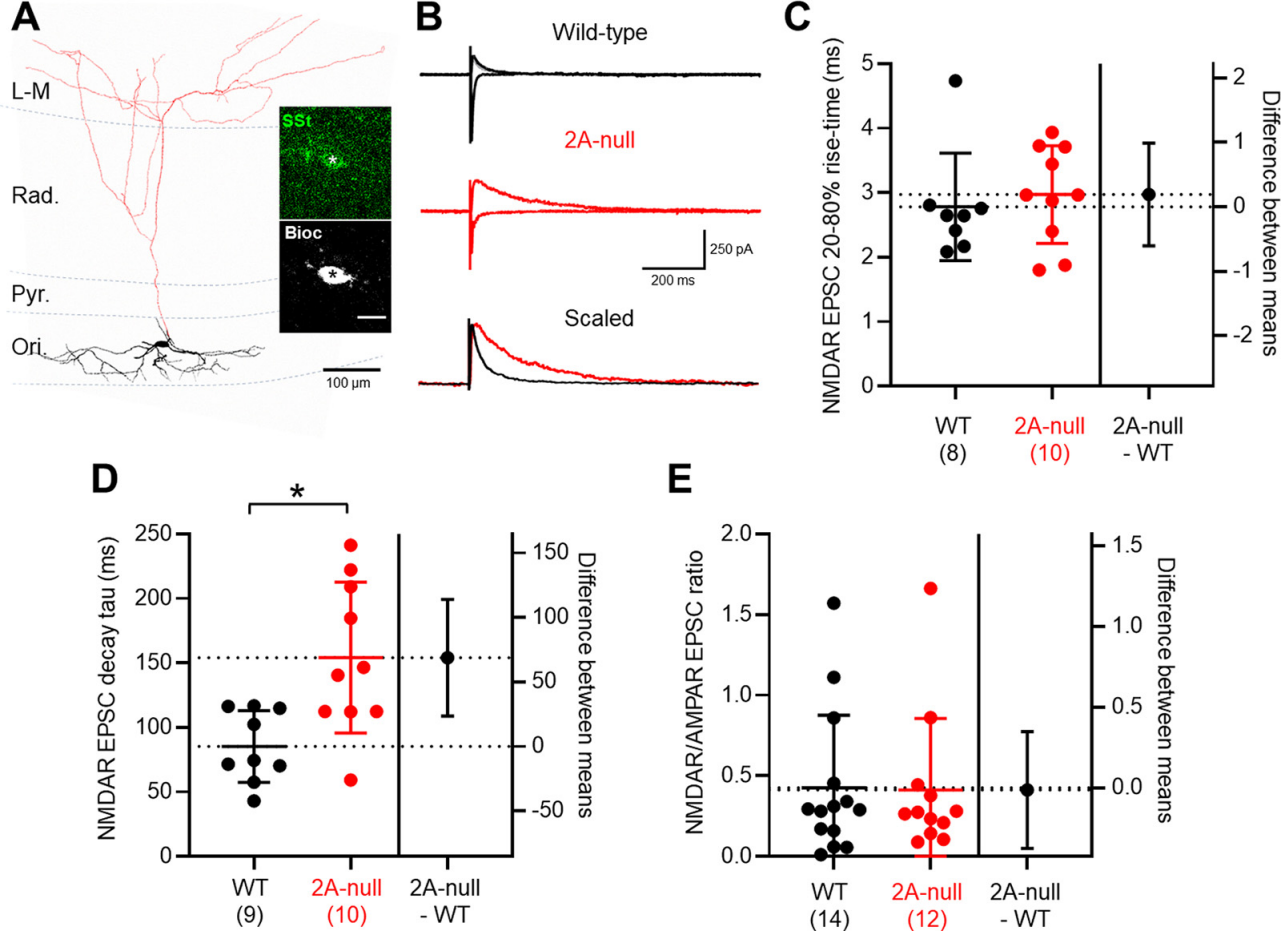
GluN2A is a major NMDAR subunit in SSt INs. ***A***, Reconstructed CA1 SSt IN shown with respect to the hippocampal layers, with somatodendritic axis confined to *stratum oriens* (black) and axons localized to *stratum lacunosum-moleculare* (L–M) in red. Inset, Immunolabelling for SSt (green) of the biocytin (Bioc)-filled somata (black and white). Scale bar, 10 μm. ***B***, Representative monosynaptic AMPAR- and NMDAR-mediated EPSCs recorded from SSt INs in wild-type (black) and 2A-null (red) rats. The scaled NMDAR-mediated EPSC (bottom) indicates the slower response in the GluN2A-null rats. ***C***, NMDAR-EPSC rise times, quantification in SSt INs, with individual cells from wild-type (black, *N* = 14 rats) and 2A-null (red, *N* = 12 rats) rats shown overlain (filled circles); fewer rats are shown for kinetic values because of the 20 pA cutoff. ***D***, Quantification of NMDAR-mediated EPSC decay time constants (tau). ***E***, NMDAR/AMPAR ratio of EPSCs elicited by *alveus* stimulation. Data are shown as the mean ± SD. *p < 0.05, Student’s *t* test.

We next asked whether the canonical feedback INs expressing SSt (Fig. 3*A*; Booker et al., 2018), possessed NMDAR-EPSCs mediated by GluN2A; all cells were included in our clear immunolabeling for SSt at the level of the soma (Fig. 3*A*, inset). The major synaptic inputs to SSt INs arise from local CA1 PyrCs (Blasco‐Ibáñez and Freund, 1995), as such we used an *alveus* extracellular stimulation to elicit monosynaptic AMPAR-EPSCs and NMDAR-EPSCs (Fig. 3*B*), as *stratum radiatum* stimulation would lead to disynaptic responses. AMPAR-EPSCs in SSt INs had comparable amplitudes of 256 ± 178 pA in wild-type rats (*n* = 17 cells, *N* = 14 rats) and 194 ± 117 pA in GluN2A-null rats (*n* = 17 cells, *N* = 12 rats; *r *=* *0.043, 62.4; CI, −62.2, 187.0; *p *=* *0.31, unpaired Student’s *t* test). Neither the 20–80% rise time (*r *=* *0.074, 0.51; CI, −0.27, 1.28; *p *=* *0.38, Mann–Whitney test) nor decay the time constant (*r *=* *0.09, 3.43; CI, −1.17, 8.02; *p *=* *0.14, unpaired Student’s *t* test) of AMPAR-EPSCs were different between genotypes.

In wild-type rats, NMDAR-EPSCs had an average amplitude of 80 ± 69 pA. NMDAR-EPSCs had a 20–80% rise time of 2.8 ± 0.8 ms (Fig. 3*C*), decay time constants of 85 ± 28 ms (Fig. 3*D*), and a NMDAR/AMPAR ratio of 0.43 ± 0.45 (Fig. 3*E*). In seven SSt INs, it was not possible to measure NMDAR-EPSC kinetics because of their failure to reach the threshold 20 pA amplitude for such measurements. The measured decay time constants of SSt INs were 30% faster than for CA1 PyrCs (*r *=* *0.21, −35.3; CI, −67.2, −3.4; *p *=* *0.032, unpaired Student’s *t* test). In GluN2A-null rats, we observed NMDAR-EPSCs of similar amplitude (68 ± 54 pA; *r *=* *0.009, −0.01; CI, −0.38, 0.35; *p *=* *0.94, unpaired Student’s *t* test), which resulted in a comparable NMDAR/AMPAR ratio of 0.41 ± 0.44 (*r *= 0.0002, −35.3; CI, −67.2, −3.4; *p *=* *0.90, Mann–Whitney test; Fig. 3*E*). These NMDAR-EPSCs had similar rise times of 3.0 ± 0.8 (*r *=* *0.015, 0.19; CI, −0.65, 1.04; *p *= 0.63, unpaired Student’s *t* test; Fig. 3*C*), but had decay time constants of 159 ± 60 ms, which was 86% longer than those of wild-type (*r *=* *0.41, 73.5; CI, 26.7, 120.2; *p *=* *0.004, unpaired Student’s *t* test; Fig. 3*D*). These properties were consistent when tested within the only morphotype identified in this study, the so-called *stratum oriens/lacunosum-moleculare* (OLM) cell, which had decay kinetics overlapping with the population average of all SSt INs (Table 1). Together, these data suggest that NMDAR-EPSCs are mediated by GluN2A subunits to a large extent in SSt INs.

**Figure 4.**
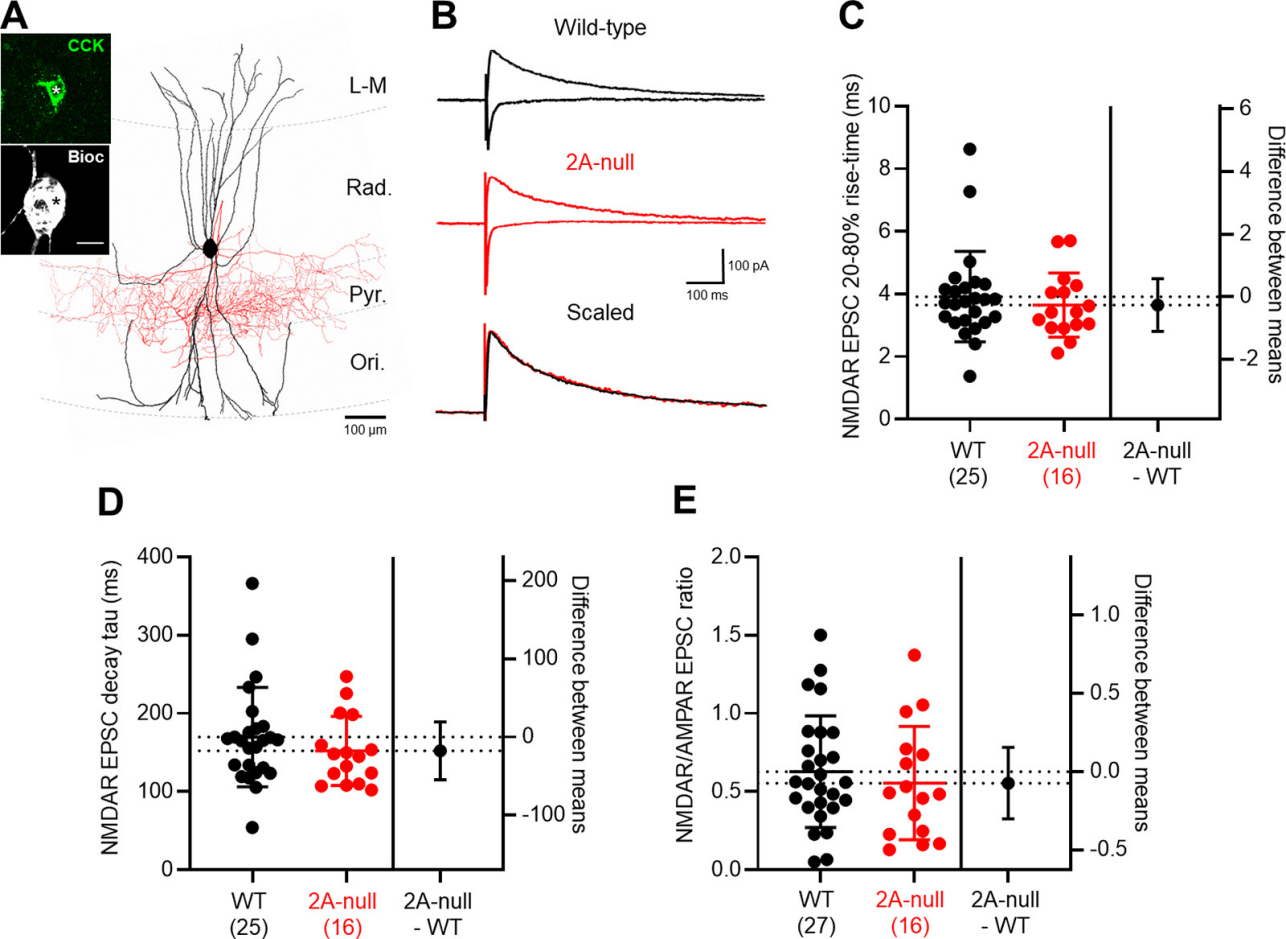
GluN2A does not significantly contribute to synaptic NMDAR-mediated EPSCs in CCK INs. ***A***, Reconstructed CCK BC with respect to the hippocampal layers, with the somatodendritic axis covering all layers (black) and axons localized to *stratum pyramidale* (red). Inset, Immunolabelling for pre-pro-CCK (green) of the IN somata (black and white). Scale bar, 10 μm. ***B***, Monosynaptic AMPAR- and NMDAR-mediated EPSCs recorded from CCK INs in wild-type (black) and 2A-null (red) rats. The scaled NMDAR-mediated EPSC (bottom) indicates no change in decay times in GluN2A-null rats. ***C***, NMDAR-EPSC rise times in CCK INs, with individual cells from wild-type (black, *N* = 26 rats) and 2A-null (red; *N* = 16 rats) shown overlain (filled circles); fewer rats are shown for kinetic values because of the 20 pA cutoff. ***D***, Quantification of NMDAR-mediated EPSC decay time constants. ***E***, NMDAR/AMPAR ratio of EPSCs elicited by *stratum radiatum* stimulation. Data are shown as the mean ± SD.

A major subtype of hippocampal INs is composed of those INs expressing the neuropeptide CCK (Booker and Vida, 2018), which likely reflect caudal ganglionic eminence INs previously identified as containing high levels of NMDARs (Matta et al., 2013) and were proposed to express GluN2A mRNA (Perszyk et al., 2016). To determine the contribution of GluN2A to synaptic currents in these neurons, we performed recordings of AMPAR-EPSCs and NMDAR-EPSCs from identified CCK INs in wild-type rats (*n* = 56 cells, *N* = 27 rats) and GluN2A-null rats (*n* = 23 cells, *N* = 16 rats), which were all confirmed with pre-pro-CCK immunolabeling (Booker et al., 2017). The cells recovered comprised BCs (*n* = 15; Fig. 4*A*), Schaffer collateral-associated/apical dendrite-associated cells (SCA/ADA, *n* = 21), and perforant path-associated (PPA; *n* = 6 cells) cells in wild-type rats; the remaining cells (*n* = 14) had their axon cut close to the cell body, preventing further classification. In GluN2A-null rats, we positively identified BCs (*n* = 7), SCA/ADA cells (*n* = 7), and PPA cells (*n* = 2); the remaining cells had a cut axon (*n* = 7) and, thus, could not be classified.

AMPAR-EPSCs recorded from CCK INs in wild-type rats had an average amplitude of 204 ± 90 pA, which was similar to that recorded in GluN2A-null rats (262 ± 129; *r *=* *0.06, −57.1; CI, −124.8, 10.56; *p *=* *0.10, unpaired Student’s *t* test). There were no observed differences in AMPAR-EPSC 20–80% rise time (*r *=* *2 × 10^−5^, 0.02; CI, −1.68, 1.73; *p *=* *0.66, Mann–Whitney test) or decay time constant (*r *=* *4 × 10^−4^, −0.29; CI, −4.59, 4.02; *p *=* *0.86, Mann–Whitney test). NMDAR-EPSCs were elicited, as for PyrCs and PV INs, by stimulation of *stratum radiatum* in the presence of picrotoxin at +40 mV (Fig. 4*B*). In wild-type rats, the average NMDAR-EPSCs had an amplitude of 99 ± 48 pA, with a rise time of 3.9 ± 1.4 ms (Fig. 4*C*), a decay time constant of 170 ± 64 ms (Fig. 4*D*), and a NMDAR/AMPAR ratio of 0.63 ± 0.36 (Fig. 4*E*). There was no apparent difference in NMDAR-EPSC properties observed between morphotypes of CCK INs (Table 1). Comparison of NMDAR-EPSCs in the GluN2A-null rats (Fig. 4*B*) did not indicate a change in rise time (3.7 ± 1.0 ms; *r *=* *0.01, −0.27; CI, −1.11, 0.57; *p *=* *0.46, Mann–Whitney test; Fig. 4*C*), an increase in decay time constant (152 ± 44 ms; *r *=* *0.02, −17.5; CI, −54.4, 19.3; *p *=* *0.27, Mann–Whitney test; Fig. 4*D*), or NMDAR/AMPAR ratio (0.55 ± 0.36; *r *=* *0.01, −0.07; CI, −0.30, 0.16; *p *=* *0.52, unpaired Student’s *t* test; Fig. 4*E*). Compared with CA1 PyrCs, the NMDAR-EPSC decay time constant was 41% longer in CCK INs (*r *=* *0.15, −49.2; CI, −88.5, −9.8; *p *=* *0.012, Mann–Whitney test), and the NMDAR/AMPAR ratio tended to be larger, albeit not significantly so (*r *=* *0.08, −0.20; CI, −0.42, 0.02; *p *=* *0.052, Mann–Whitney test). Together, these data suggest that CCK INs possess a high level of NMDARs with markedly different kinetics from CA1 PyrCs and appear to lack GluN2A subunits.

### Differential ifenprodil sensitivity of NMDARs indicates divergent GluN2B expression

**Figure 5.**
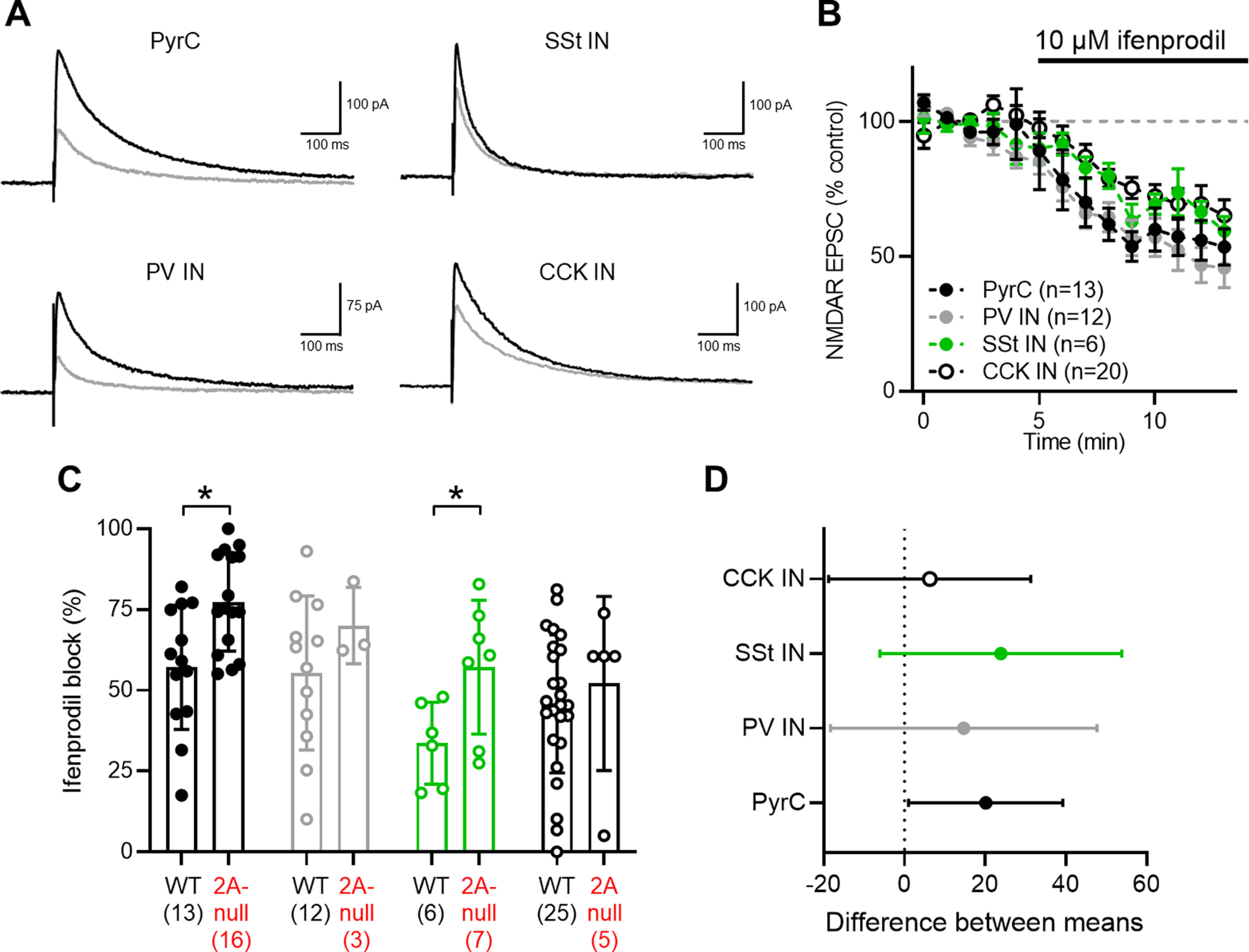
GluN2B-containing NMDARs differentially contribute to EPSCs in identified hippocampal neurons. ***A***, Representative NMDAR-mediated EPSCs recorded at +40 mV in the presence of CNQX at (black traces) or following 10 min wash-in of 10 μm ifenprodil (gray traces) from wild-type rats (WT) for the different cell types identified. ***B***, Time course of ifenprodil wash-in (black bar) for CA1 PyrCs (open circles), PV INs (filled black circles), SSt INs (gray circles), and a subset of CCK INs (green circles), measured as a percentage of control EPSCs per minute, compared with 100% at baseline (dashed black line). ***C***, Quantification of ifenprodil block over the last 2 min for the different cell classes identified in wild-type and GluN2A-null neurons (2A-null); number of cells tested is shown in parenthesis. ***D***, Estimation plot showing the difference in ifenprodil block between wild-type and GluN2A-null cells, plotted as the difference between means ± 95% confidence interval. Data are shown as the mean ± SD. *p < 0.05, paired *t* tests.

The data presented so far indicate that in CA1 PyrCs, PV INs and SSt INs GluN2A subunits contribute to synaptic NMDAR-EPSCs, but that effects of GluN2A loss are largely absent at functional synaptic receptors on CCK INs. We next sought to determine the relative expression of GluN2B subunits, through their pharmacological blockade with the selective GluN2B antagonist ifenprodil (10 μm; Williams, 2001; Lei and McBain, 2002). In all cell types tested, we observed a degree of ifenprodil sensitivity when applied to pharmacologically isolated NMDAR-EPSCs (Fig. 5*A*). Blockade of NMDAR-EPSCs mediated by ifenprodil was use-dependent, as previously described (Kew et al., 1998), and developed slowly in all cells tested (Fig. 5*B*). In CA1 PyrCs, following a 10 min wash-in, ifenprodil produced a strong 55 ± 21% block of native NMDAR-EPSCs, reducing the average amplitude from 136 ± 78 to 54 ± 30 pA (*N* = 13 rats; *r *=* *0.60, −82.4; CI, −137.4, −27.4; *p *=* *0.009, paired Student’s *t* test; Fig. 5*C*), consistent with the presence of GluN2B receptor subunits (Gray et al., 2011; Yi et al., 2019), but a greater block than for triheteromeric receptors alone (Hansen et al., 2014). This ifenprodil block in CA1 PyrCs was stronger in GluN2A-null rats at 83 ± 13% (*N* = 8 rats; *r *=* *0.48, −25.2; CI, −48.7, −1.7; *p *=* *0.039, paired Student’s *t* test), which is consistent with a loss of GluN2A subunit containing triheteromeric receptors (Hansen et al., 2014). In PV INs, the average NMDA-EPSC amplitude was blocked by 55 ± 24%, from 88 ± 65 to 39 ± 33 pA (*N* = 12 rats; *r *= 0.61, −49.5; CI, −79.4, −19.6; *p *=* *0.005, paired Student’s *t* test; Fig. 5*C*). PV INs in GluN2A-null rats (*N* = 3 rats) displayed a 70 ± 12% ifenprodil block, which was statistically not different from that in wild-type rats (*r *=* *0.09, 15.0; CI, −17.7, 47.6; *p *=* *0.34, unpaired Student’s *t* test). In contrast, SSt INs displayed a 34 ± 13% block, with NMDAR-EPSCs reduced from 145 ± 46 to 107 ± 42 pA (*N* = 5 rats; *r *=* *0.55, −37.3; CI, −83.7, 9.1; *p *=* *0.094, paired Student’s *t* test), which increased to a 57 ± 14% block in the GluN2A-null rats (*N* = 6, *r *=* *0.47, 23.1; CI, 5.9, 40.3; *p *=* *0.014, unpaired Student’s *t* test). CCK INs displayed a 42 ± 23% block from 126 ± 60 to 64 ± 38 pA (*N* = 12 rats; *r *=* *0.52, −30.6; CI, −47.4, −13.8; *p *=* *0.002, paired Student’s *t* test). In GluN2A-null rats (*N* = 3), ifenprodil produced a block of 57 ± 10%, which was not different from that of wild-type rats (*r *=* *0.07, 15.2; CI, −13.4, 43.8; *p *=* *0.28, unpaired Student’s *t* test). Comparison of the ifenprodil block between cell types revealed that there was no substantial difference between cell types in wild-type rats (*F* = 1.864, *p* = 0.15, one-way ANOVA; Fig. 5*C*). In all but CCK INs, the degree of ifenprodil block tended to be greater in the GluN2A-null rat, indicating a greater contribution of this subunit to synaptic NMDARs (Fig. 5*D*). These data suggest that GluN2B sensitivity is broadly equivalent among PyrCs, PV, SSt, and CCK INs, and that GluN2B subunits contribute to heterotrimeric NMDARs in all cell classes.

### NMDARs in CCK INs are GluN2D containing and contribute to LTP induction

**Figure 6.**
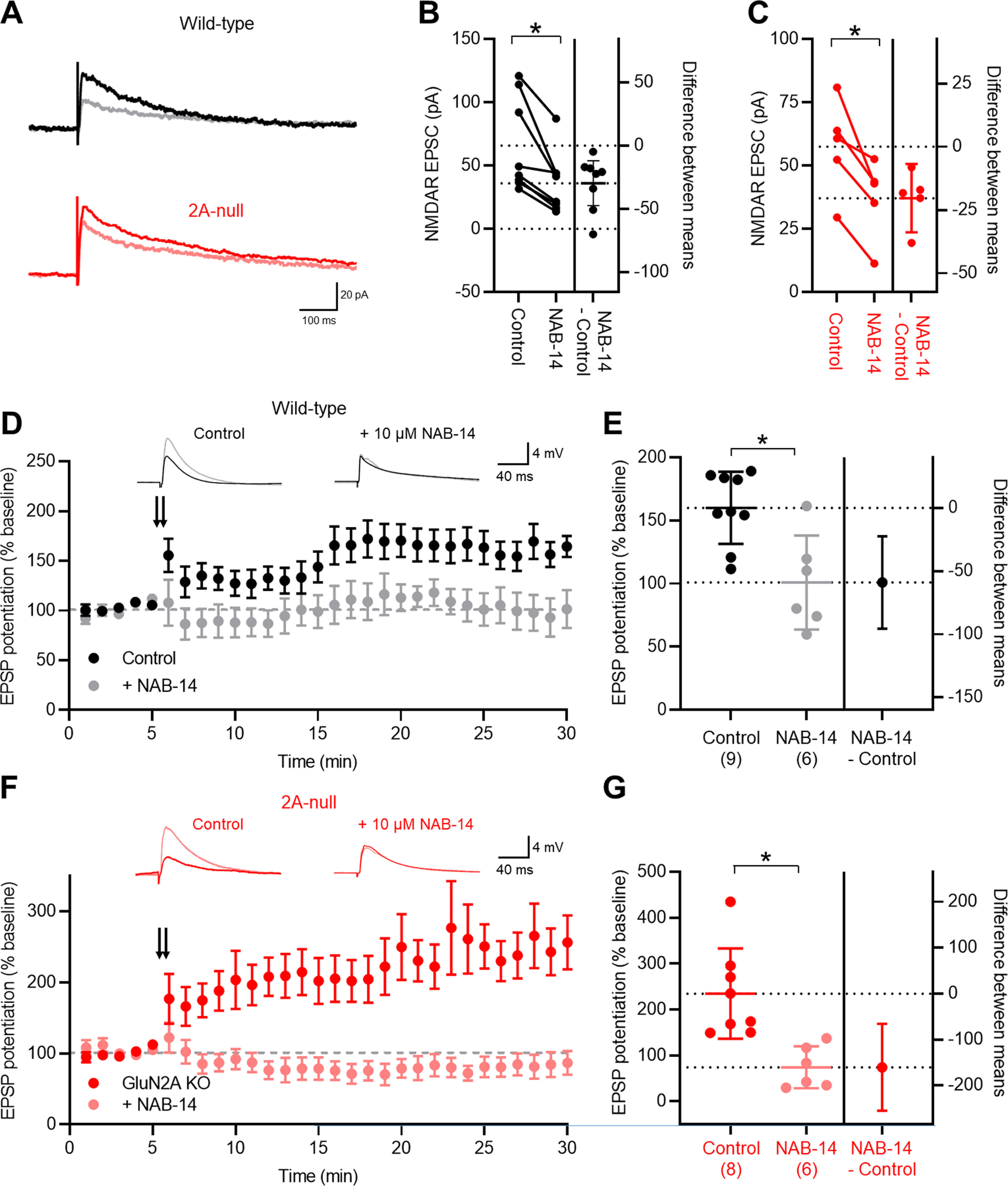
NMDAR-EPSCs in CCK INs are mediated by GluN2D, which is required for LTP induction. ***A***, Representative NMDAR-mediated EPSCs recorded in CCK INs at +40 mV in the presence of CNQX at (black and red traces) or following 10 min wash-in of the GluN2D-negative allosteric modulator NAB-14 (10 μm; gray and pink traces). ***B***, Plot of NMDAR-EPSC amplitudes before and after NAB-14 application in CCK INs. ***C***, Quantification of NMDAR-EPSC amplitudes in CCK INs from GluN2A-null rats before and after NAB-14 application. ***D***, Time course of EPSP amplitude in CCK INs from wild-type rats measured from −70 mV current clamp, following 2× 100 Hz stimulation (double arrow) measured under control conditions (black circles) and in the presence of 10 μm NAB-14 (gray circles). Representative traces are shown above the chart, showing EPSP at baseline (black traces) and 25 min after LTP induction (gray traces). ***E***, Quantification of LTP, reported as the percentage change in EPSP amplitude from baseline (dashed line) for wild-type CCK INs. Control LTP recordings (black circles; *n* = 9 cells; *N* = 7 rats) and those performed in the presence of 10 μm NAB-14 (gray circles; *n* = 5 cells; *N* = 5 rats) are shown. The number of tested rats is shown in parentheses. ***F***, LTP induction in CCK INs from GluN2A-null rats according to the same scheme as in ***D***. Data are shown for control recordings (red circles) and in the presence of NAB-14 (pink circles). Traces of each treatment are above the chart showing data at baseline (red) and after LTP induction (pink traces). ***G***, Quantification of LTP induction in GluN2A-null rats under control (red; *n* = 6 cells; *N* = 5 rats) and in the presence of NAB-14 (pink; *n* = 5 cells; *N* = 4 rats). Data are shown as the mean ± SD. *p < 0.05, from paired (***B***) and unpaired (***D***, ***F***) Student’s *t* tests.

From the data we have shown so far, CCK IN NMDAR-EPSCs appear to be unaffected by the loss of GluN2A. Furthermore, CCK INs express high levels of GluN2D mRNA 7 (Perszyk et al., 2016), and *stratum radiatum* INs also possess NMDAR-EPSCs sensitive to GluN2C/D modulation (Perszyk et al., 2016; Swanger et al., 2018; Yi et al., 2019). To determine whether GluN2D-containing receptors specifically contribute to NMDAR-EPSCs in CCK INs, we used the GluN2C/D-negative allosteric modulator NAB-14 (Swanger et al., 2018). As GluN2C is minimally expressed in hippocampal neurons (Wenzel et al., 1997; Perszyk et al., 2016), it is likely that the effects of NAB-14 can be attributed to NMDAR-containing GluN2D subunits. Bath application of NAB-14 (10 μm) to recordings of pharmacologically isolated NMDAR-EPSCs substantially reduced their amplitude in CCK INs from wild-type and GluN2A-null rats (Fig. 6*A*). Following a 10 min wash-in of NAB-14, NMDAR-EPSCs were reduced by 65% (*n* = 13 cells; *r *=* *0.53, −75.2; CI, −119.9, 30.5; *p *= 0.0002, Wilcoxon test), and by 37% in GluN2A-null rats (*n* = 5 cells; *r *=* *0.81, −20.4; CI, −33.9, −6.8; *p *=* *0.014, paired Student’s *t* test), which was not statistically different between genotypes (*r *=* *0.19, −23.7; CI, −50.0, 2.6; *p *=* *0.074, unpaired Student’s *t* test). Bath application of NAB-14 did not alter the 20–80% rise time of NMDAR-EPSCs in either wild-type (*r *=* *0.007, 0.30; CI, −2.1, 2.7; *p *=* *0.79, paired Student’s *t* test) or GluN2A-null CCK INs (*r *=* *0.097, −0.18; CI, −0.92, 0.57; *p *=* *0.55, paired Student’s *t* test). As expected from a selective block of GluN2D-containing receptors, the decay time constants of NMDAR-EPSCs were shortened by 42% for wild-type (*r *=* *0.61, −109.7; CI, −168.3, −51.0; *p *=* *0.002, paired Student’s *t* test), and by 50% for CCK INs in GluN2A-null rats (*r *=* *0.67, −98.5; CI, −194.3, −2.7; *p *=* *0.046, paired Student’s *t* test); the magnitude of this effect was not different between genotypes (*r *=* *0.006, −3.8; CI, −30.3, 22.7; *p *=* *0.046, paired Student’s *t* test). Together, these data reveal that NMDAR-EPSCs in CCK INs result from activation of NMDARs composed of GluN2D and GluN2B, but not GluN2A subunits.

NMDARs are known to mediate Hebbian LTP, in both PyrCs (Malenka, 1991) and INs (Lamsa et al., 2005, 2007). Indeed, GluN2A/B-containing receptors have been shown to induce LTP (Berberich et al., 2005), with the GluN2A subunit presumed to be requisite for LTP induction (Liu et al., 2004). Given the absence of GluN2A from CCK INs, we asked whether the presence of GluN2D-containing NMDARs was sufficient to induce the Hebbian form of LTP present in these INs (Lamsa et al., 2007). In whole-cell recordings from CCK INs, performed in current clamp, monosynaptic EPSPs were evoked from putative Schaffer collateral afferents in *stratum radiatum* in the presence of picrotoxin. The average amplitude of these EPSPs was 5.4 ± 1.9 mV in wild-type CCK INs (*N* = 9 rats). Following 5 min of baseline recording, LTP was induced by delivering 2× 100 Hz trains of stimuli (duration, 1 s) to the stimulated pathway, the EPSP amplitude was then measured 25 min after induction (Fig. 6*C*). In control recordings from wild-type CCK INs, this protocol resulted in potentiation of the EPSP to 59 ± 30% increase from control EPSP at 25 min (*r *=* *0.76, 3.4; CI, 1.9, 5.0; *p *= 0.001, paired Student’s *t* test; Fig. 6*D*,*E*). In recordings from wild-type rats (*N* = 6 rats), NAB-14 (10 μm) was preapplied (10 min before recording), then CCK INs were recorded. The baseline EPSPs in the presence of NAB-14 had an average amplitude of 6.5 ± 3.4 mV, which was not different from that of control EPSPs (*r *=* *0.05, 1.1; CI, −1.9, 4.1; *p *=* *0.45, unpaired Student’s *t* test). Following the same induction as for control recordings, in the presence of NAB-14, little to no potentiation of the EPSP amplitude (6 ± 37% increase) was observed at 25 min after tetanization (*r *=* *0.004, 0.14; CI, −2.2, 2.5; *p *=* *0.89, paired Student’s *t* test; Fig. 6*D*,*E*), which was markedly lower than the potentiation observed in control recordings (*r *= 0.46, −57.7; CI, −95.0, −20.3; *p *=* *0.005, unpaired Student’s *t* test). To confirm that LTP in CCK INs was independent of GluN2A, we performed the same recordings in GluN2A-null rats. Following the same induction paradigm, we observed sustained synaptic potentiation in CCK INs from the GluN2A-null rat, with a 134 ± 98% increase in EPSP amplitude under control conditions (*N* = 8 rats; *r *=* *0.53, 7.4; CI, 1.1, 13.6; *p *=* *0.008, Wilcoxon test; Fig. 6*F*,*G*). Contrary to LTP being reduced in the absence of GluN2A, the magnitude of EPSP potentiation was 48% greater in CCK INs of the GluN2A-null rat (*r *=* *0.25, 76.0; CI, 3.1, 149.0; *p *=* *0.042, unpaired Student’s *t* test). In the presence of NAB-14, there was no potentiation of the EPSP when measured at 25 min in CCK INs from the GluN2A-null rats (*N* = 6 rats; 36 ± 46%; *r *=* *0.092, −0.56; CI, −2.6, 1.5; *p *=* *0.51, paired Student’s *t* test; Fig. 6*F*,*G*). These data reveal that LTP at Schaffer collateral synapses onto CCK INs in part depend on GluN2D-containing NMDARs, but not GluN2A-containing NMDARs.

## Discussion

In the current study, we provide evidence for GluN2A-specific modulation of synaptic NMDAR-mediated currents in hippocampal INs and PyrCs. We show that NMDAR-EPSCs in PV INs closely resemble those of CA1 PyrCs, in terms of kinetics, synaptic contribution, and GluN2A/2B composition. The closely related SSt IN cell type displays NMDAR-EPSCs that are typically faster and likely composed largely of GluN1/2A or GluN1/2A/2B receptors. Finally, we show unequivocally that CCK INs lack a prominent GluN2A component to their NMDAR-EPSCs; rather, their synaptic NMDARs are likely composed of GluN1/2B/2D triheteromeric receptors based on ifenprodil and NAB-14 sensitivity. Finally, we confirm that these NMDARs in CCK INs are required to induce a Hebbian form of LTP. Together, the data we present provide further evidence that different IN subtypes display divergent synaptic NMDAR pharmacology.

### Functional identification of GluN2 subunits in hippocampal neurons

CA1 PyrCs have been postulated to express both GluN2A/B triheteromeric receptors and GluN2A or GluN2B diheteromeric receptors in mature neurons (Al-Hallaq et al., 2007; Traynelis et al., 2010; Gray et al., 2011; Tovar et al., 2013). In this study, we provide evidence that CA1 PyrCs likely express at least both GluN2B diheteromeric and GluN2A/B triheteromeric receptors at functional synapses in late juvenile rats. NMDAR-mediated EPSCs were significantly slowed in both rise and decay phase following the loss of GluN2A subunits, but did not have altered amplitudes. The observed 50–60% block produced by ifenprodil in wild-type PyrCs is consistent with the pharmacology of GluN1/2A/2B triheteromeric NMDARs observed in both heterologous cell lines (Erreger et al., 2005; Hatton and Paoletti, 2005; Marwick et al., 2019) and also in cultured dissociated neurons (Martel et al., 2009; McKay et al., 2012). Indeed, the increased block observed in the GluN2A-null rat is consistent with a switch to GluN1/2B diheteromeric receptors at synapses (Gray et al., 2011; McKay et al., 2012). Nevertheless, ifenprodil has been suggested to block GluN2A-containing receptors to some degree in cultured neurons (Kew et al., 1998; Williams, 2001); however, the IC_50_ in NMDARs comprising only the Glu2A receptor is an order of magnitude higher than we used in this study (Avenet et al., 1996). Indeed, the ifenprodil block we observe is greater in the absence of GluN2A, consistent with a selective effect at GluN2B-containing NMDARs. Furthermore, a range of ifenprodil concentrations up to and including 10 μm have been used in *ex vivo* slice preparations, with overall findings consistent with GluN2B selective effects (Lei and McBain, 2002; Matta et al., 2013). Together, these data show in late juvenile rats that synaptic NMDA receptors are likely composed of a combination of GluN2A/B triheteromeric and GluN2B diheteromeric receptors, which accounts for the slower kinetics and tendency toward reduced NMDAR/AMPAR ratios at Schaffer collateral synapses in these neurons.

The role of NMDARs in the signaling, plasticity, and excitability of hippocampal INs is poorly understood, and that of the contribution of different NMDAR subunits to these functional properties even less so (Moreau and Kullmann, 2013; Akgül and McBain, 2016; Booker and Wyllie, 2021). Many studies have suggested that PV INs likely possess NMDAR-mediated EPSCs with approximately four-fold lower amplitudes compared with AMPAR-mediated EPSCs (Koh et al., 1995; Korotkova et al., 2010; Matta et al., 2013). In the neocortex, such NMDAR-EPSCs possess a GluN2A component in the neocortex (Picard et al., 2019) and display a low GluN2B content in the hippocampus of juvenile mice (Matta et al., 2013). We show that CA1 PV INs possess NMDARs of similar composition to those in CA1 PyrCs based on recordings in the GluN2A-null rat and in the presence of ifenprodil. These NMDAR-EPSCs have faster kinetic properties than neighboring CA1 PyrCs (Gray et al., 2011; see also current data), which in the absence of reduced synaptic amplitudes in the GluN2A-null rat may relate to the altered dendritic properties of PV INs compared with CA1 PyrCs (Nörenberg et al., 2010) or altered NMDA effects on such (Camiré and Topolnik, 2014). Our finding that the amplitude of NMDARs-EPSCs in PV INs is comparable to that of CA1 PyrCs, with similar effects of GluN2A loss and GluN2B blockade, offers an explanation for the presence of readily inducible Hebbian or anti-Hebbian LTP in the PV and SSt IN classes, reliant on the presence of NMDARs or calcium-permeable AMPARs, respectively (Laezza et al., 1999; Perez et al., 2001; Lamsa et al., 2005; Kullmann and Lamsa, 2007; Szabo et al., 2012; Billingslea et al., 2014). Indeed, activating these NMDARs likely recruits signaling cascades similar to those in PyrCs (Berberich et al., 2005; Gray et al., 2011), with a defined role in plasticity induction (Camiré and Topolnik, 2014). Our data suggest that, in rats, NMDAR-EPSCs may be mediated by a greater proportion of GluN2B-containing receptors than in mice, given the differential ifenprodil sensitivity observed (Matta et al., 2013), although this has some potential caveats (see above). The presence of large NMDAR-EPSCs, which are similar to CA1 PyrC synaptic NMDARs, may indicate that NMDARs underpin similar roles in terms of dendritic integration in PV INs (Branco et al., 2010; Cornford et al., 2019). While SSt INs share a common developmental origin with PV INs (Tricoire et al., 2011; Chittajallu et al., 2013) and also coexpress PV (Jinno and Kosaka, 2000; Booker et al., 2018), our data indicate that through their differentiation they express functional NMDAR-EPSCs that are distinct from those of PyrCs and other PV IN morphotypes. While the single-channel conductance of NMDARs in OLM cells is similar to that of CA1 PyrCs (Hájos et al., 2002) and their dendritic filtering is rapid (Martina et al., 2000) and similar to that in PV INs (Nörenberg et al., 2010), our data indicate there are fundamental differences in the relative contribution of NMDAR subunits to EPSCs in these cells. In particular, it is plausible that SSt INs possess a more uniform population of GluN2A/2B triheteromeric receptors, first because of the ∼30% block with ifenprodil (Hansen et al., 2014) and the longer decay kinetics of NMDAR-EPSCs we observe in the GluN2A-null rats. It has been shown in mice that GluN2D is expressed in PV and SSt INs in adult mice (Standaert et al., 1996; Engelhardt et al., 2015); however, given the rapid kinetics and high sensitivity of NMDAR-EPSCs to GluN2A loss and ifenprodil, our data suggest that this NMDAR subunit may not contribute to synaptic currents in older rats. Indeed, it is plausible that GluN2D is expressed in rat neurons but may contribute to nonsynaptic mechanisms. As LTP in putative SSt INs is dependent on NMDARs (Ouardouz and Lacaille, 1995), these receptors likely fulfill the typical role of NMDARs in LTP induction (Berberich et al., 2005) in this fundamental feedback interneuron subtype. One major consideration to the function of NMDARs in INs is that GluN2A subunits undergo a developmental increase in expression in PyrCs and INs alike (Flint et al., 1997; Martel et al., 2009; McKay et al., 2012; Matta et al., 2013; Wyllie et al., 2013). How this contributes to the circuit recruitment of INs has not been fully explored other than in a few IN subtypes (Matta et al., 2013). Indeed, whether or not GluN2D subunits undergo such a developmental trajectory in hippocampal INs, or indeed whether GluN2A subunits possess a delayed maturation in CCK INs remains unexplored.

We observed NMDAR-mediated currents in identified CCK IN cell types that were larger and slower than those observed in CA1 PyrCs. This is in good agreement with the presence of GluN2B/2D NMDARs, as described in *stratum radiatum* INs (Perszyk et al., 2016; Yi et al., 2019) and in GluN2D-containing receptors more generally (Wyllie et al., 1996; Misra et al., 2000; Jones and Gibb, 2005; Logan et al., 2007; Brothwell et al., 2008). The sensitivity of NMDAR-EPSCs in CCK INs to NAB-14 confirms the presence of GluN2C/D receptor subunits in synaptic receptors, with a reduced sensitivity to ifenprodil (Yi et al., 2019) compared with GluN2A/2B triheteromeric receptors. However, as GluN2C is not present in the hippocampus, the predominant receptor target is likely GluN2D-containing NMDARs. The absence of GluN2A-mediated EPSC effects is in good agreement with that in recordings performed in non-identified *stratum radiatum* INs, where a glycine-sensitive GluN2A antagonist did not alter NMDAR-EPSCs (Yi et al., 2019). Given that LTP was strongly attenuated by NAB-14 and the lack of CP-AMPAR-mediated LTP (Szabo et al., 2012) in identified CCK INs, our results support previous findings suggesting a central role of NMDARs in synaptic plasticity in these INs (Lamsa et al., 2007). Similarly, as LTP was readily induced in CCK-INs, we confirm previous work showing that GluN2D-containing NMDARs aid this induction (Hrabetova et al., 2000). Together, these data support the idea that GluN2A-containing NMDARs are not required for the induction of LTP in CCK INs (Berberich et al., 2005), but rather it is GluN2D-containing NMDARs that contribute to LTP expression.

Overall, our data extend the previous findings of the study by Perszyk et al. (2016), in which the reported mRNA corresponding to GluN2A, GluN2B, and GluN2D was abundant in each of the neurochemical cell types we have assessed. For example, this study shows that GluN2A receptor subunits do not appear to contribute to synaptic NMDAR-mediated currents in CCK-INs. While our data do not preclude the possibility that other NMDAR subtypes exist in all of the neuron classes tested, they indicate that such roles would be confined to other (non-synaptic or non-ionotropic) functions, such as extrasynaptic modulation of plasticity (Ivanov et al., 2006), metabotropic modulation of inhibitory receptors (Chalifoux and Carter, 2010; Guetg et al., 2010), or mediating tonic excitation (Hanson et al., 2019). Furthermore, as we have only examined the major synaptic inputs to different classes of CA1 PyrCs and INs, synapse-specific effects of receptor composition may exist (Arrigoni and Greene, 2004; von Engelhardt et al., 2008). Indeed, recent data suggest that cell type-specific inputs may lead to within-cell differences in synaptic composition of NMDARs (Lei and McBain, 2002). Our data suggest that most synaptic NMDARs contain a mixed contribution of receptor subtypes, which provides the tantalizing prospect that synapse and extrasynaptic heterogeneity of NMDARs exists to give rise to functional divergence. The data we present suggest that further work is required to disentangle the contributions of different NMDAR subtypes to synaptic and extrasynaptic functions in hippocampal INs.

### Functional ramifications

Given the large heterogeneity in GluN2 subunit-specific effects on NMDAR-EPSCs in hippocampal INs, there is a wide variety of potential ramifications for human disease. First, given that GluN2A subunits contribute significantly to PV and SSt IN synaptic NMDARs, this heterogeneity emphasizes the need to further elucidate the role of these neurons in epileptic syndromes, as the GluN2A subunit may be a critical determinant of some forms of epilepsy (Marwick et al., 2019). Furthermore, as GluN2D *de novo* mutations have also been linked to epileptic encephalopathies (Li et al., 2016; Camp and Yuan, 2020), and given the clear role of this subunit in CCK IN synaptic function, the heterogeneity may explain the selective loss of these neurons in some forms of experimental epilepsy (Wyeth et al., 2010; Sun et al., 2014). These data suggest potential therapeutic avenues by which NMDAR function is selectively modulated in a brain region and in a cell type-specific manner. Furthermore, NMDARs contribute to the spike coupling of INs to ongoing synaptic activity (Matta et al., 2013). How divergent NMDAR kinetic properties between different IN subtypes contribute to ongoing circuit activity remains unknown, but will likely have direct ramifications for local information processing.

In summary, we provide clear evidence for GluN2A, GluN2B, and GluN2D subunit-containing NMDARs in neurochemically and morphologically defined INs and PyrCs of the rat. These findings provide insight into the mechanisms of synaptic plasticity generation in CCK INs, which rely on GluN2B- and GluN2D-containing, but not GluN2A-containing, NMDARs. These data are important to our understanding of how different IN classes integrate synaptic information.

## Synthesis

Reviewing Editor: Isabel Perez-Otaño, Instituto de Neurociencias Decisions are customarily a result of the Reviewing Editor and the peer reviewers coming together and discussing their recommendations until a consensus is reached. When revisions are invited, a fact-based synthesis statement explaining their decision and outlining what is needed to prepare a revision will be listed below. The following reviewer(s) agreed to reveal their identity: Chris McBain.

The paper was considered of significant interest by both reviewers, and is a nice addition to the NMDAR field given our limited knowledge of the roles in INs.

REVIEWER´S COMMENTS

Reviewer #1

In this manuscript titled “Contribution of N-methyl D-aspartate receptors to synaptic function in rat hippocampal interneurons", the authors present data that suggest the GluN2A subunit of the NMDA receptor makes unequal contributions to synaptic transmission across various types of interneurons in the CA1 subfield of the rat hippocampus. Using a CRISPR-Cas9 knockout rat model, the researchers delete the Grin2a gene and use the EPSC time course as a readout of GluN2A’s influence on excitatory tone onto PV-, CCK-, and SST-expressing interneurons. Moreover, the authors conclude that each of these interneuron subtypes utilizes GluN2B-containing NMDA receptors for synaptic transmission via ifenprodil inhibition of the evoked EPSC and that the GluN2D subunit expression may have important implications for LTP onto CCK-expressing interneurons.

Overall, the rationale for this work is sound, and the information gained from this study may help future drug development. Interneurons and the inhibitory network can be dysfunctional in a host of neurological conditions and thus their modulation may prove therapeutically useful. However, there are several concerns that should be addressed before publication.

Major concerns:

1.There does not appear to be any justification for the number of replicates completed in the manuscript, ranging from as little as 4 animals (5 cells) up to 24 animals (52 cells). It almost appears as if the authors arbitrarily chose the number of replicates needed for experiment. Without more explanation, it is not possible to ascertain why certain measures, such as the NMDA receptor-mediated EPSC decay time for CCK-expressing interneurons required 24 animals (52 cells) while an analogous experiment, the tau decay for pyramidal cells, required far fewer. There also is no mention of power analysis to substantiate selection of number of observations.

2.The authors report that EPSCs recorded from WT pyramidal cells at 30 degrees C had a weighted tau of 139 ms and that GluN2A-KO mice had a prolonged decay time (211 ms). These data agree with previously published data. However, the weighted tau decay times for WT PV-expressing interneurons is reported as 73 ms and SST-expressing interneurons as 59 ms. These decay values do not match previously published data, nor do they align with the idea that GluN2D-containing NMDA receptors have been reported on both of these interneuron subtypes. For example, Cornford et. al. 2019 (PMID: 31657720) report NMDA receptor-mediated EPSCs, evoked via Schaffer collateral stimulation at 30 degrees C, to have an average weighted tau decay time of roughly 150 ms. In this manuscript, the authors report three PV-expressing interneurons to have a weighted tau decay time of less than 50 ms, and we were unable to find any report of similar values in the literature. These concerns are also shared for SST-expressing interneurons. The authors need to compare their results to those in the literature in order to provide both context for their results and to ensure that the recordings/analyses are not subject to technical errors. For example, some of their measured NMDA/AMPA ratios are near zero, raising a question as to whether the briefest taus were subject to artifacts and noise, possibly as a result of small amplitude. Could the authors reanalyze the deactivation data with strict cut off for response amplitude to ensure the results were not influenced by noise or baseline drift for the smallest responses?

3.The data in the time course plot in Figure 5B does not seem consistent with the bar graph in Figure 5C. The CCK and SST plots are on top of each other in the time course plot (Figure 5B) but show a 20% difference in the bar graph representation (Figure 5C). The time course plot suggests there are two groups, PC/PV and CCK/SST, with clear and large differences, but the biggest difference in the bar graph appears to only differentiate SST- and CCK-expressing interneurons.

4.A baseline period of only five minutes may be too short for a plasticity experiment, with most studies reporting 15-20 minutes. Figure 6C almost looks as if the response amplitude is trending upward prior to the LTP stimulus, raising a question of whether the recording was stable before LTP induction.

5.Figure 6A suggests that NAB-14 is less effective in blocking NMDAR responses in the 2A-null mice but there appears to be no quantification of NAB-14 effects on EPSCs from 2A-null mice.

Minor concerns:

1.The introduction and layout of the manuscript do not provide a clear explanation of the importance and design of the LTP experiments for only one interneuron subtype.

2.The discussion does not really place the results in the context of what is known, and also misses an opportunity to identify any weaknesses or caveats in the manuscript. For example, the lack of GluN2A’s participation in excitatory transmission onto CCK-expressing interneurons is not really discussed. There is also no discussion of the temporal timing of GluN2A expression, a topic relevant for development. The authors should explicitly comment that the study only probes feedforward inhibition, neglecting synaptic connections utilized in feedback inhibition. The authors should also ensure they clearly articulate for those not in the field they have focused on only a couple interneuron types, and ensure they do not leave a reader with the impression that there only three main neurochemical markers for interneurons in the CA1 subfield.

3.10 M ifenprodil is not the optimal concentration from which to draw conclusions about GluN2B involvement. 3 M would have been better, since at 10 M there can be some non-selective block of GluN2A by ifenprodil. This should be raised as a caveat.

4.The lack of a change in the time course of NMDA receptor-mediated EPSCs following NAB-14 treatment could occur if most receptors at the synapse were triheteromeric receptors that contained either GluN2A/GluN2D or GluN2B/GluN2D, with no GluN2D diheteromeric receptors. In this situation, GluN2A or GluN2B could dominate the deactivation, and NAB-14 would simply reduce the number of active receptors. One would only expect a change in the time course if removal of GluN2D-containing receptors revealed another NAB-14-resistant population of NMDA receptors with a different response time course.

5.The authors should consider the possibility that the lack of LTP in NAB-14 is due only to reduced NMDA activity overall and not something specific about the presence of GluN2D, and thus perhaps a more appropriate conclusion would be that GluN2D “contributes” to LTP and not that GluN2D “is required” for LTP.

6.The manuscript mentions that series resistance was monitored during recording of evoked EPSCs and cells were excluded if series resistance changed by more than 20%. However, no further details are provided. It would be nice to see exactly how this was done and what series resistance ranges were.

7.The pulse stimulation duration is not mentioned.

8.An explanation of the intensity of the voltage used to stimulate the slice is lacking and it is unclear how the stimulus intensity was selected. Was an input-output curve generated prior to initiation of the experiment?

9.The immunohistochemical staining of the sample images provided for SST- and CCK-expressing interneurons is weak, with barely discernable somas. Perhaps these slices could benefit from less fixation or antigen retrieval.

10.There is no mention of a correction for family-wise error, although two measures were statistically compared from the same recording (AMPAR/NMDAR amplitude ratio, decay time).

11.The authors only use male rats and should provide an explanation of why females were not included.

12.The criteria for termination of the experiment was described as baseline responses being unstable but the criteria for what is stable or not is not defined.

Reviewer #2

This is a very nice and straightforward examining the role of NMDAR subtypes on identified inhibitory interneurons that takes advantage of the recently reported GluN2A null rat. The authors demonstrate a differential role for GluN containing NMDARs and highlight that PV and SST interneurons differ in their NMDAR composition from putative CCK-containing interneurons in the CA1 subfield of the rat hippocampus. The data are clear and convincing and the conclusions are warranted and supported by the data. I have only a few minor comments that I hope the authors will find useful in revising their manuscript.

The data from both PV and SST-containing interneurons shows that a lack of GluN2A yields NMDAR meditated currents of similar amplitude to wildtype but with longer apparent decay kinetics as would be expected with the elimination of GluN2A. The lack of change in both the amplitude of the NMDAR mediated current and the AMPA/NMDAR ratio is surprising, and suggests that receptors containing GluN2A are certainly involved in this EPSC but other subunits remain and/or compensate for the lack of GluN2A. In their summing up of this data set (eg line 323 page 11) the authors should conclude that GluN2A likely contributes to the observed NMDARs EPSCs but it certainly doesn’t “mediate” the synaptic current since there appears to be sufficiently functional NMDARs in its absence. Semantics maybe, but an important clarification nonetheless.

Page 12. How certain can the authors be that all of the radiatum cells from which recordings were made were in fact CCK-positive cells. There are many cell types that reside in the st. radiatum in addition to the CCK-cell population. How many recovered cells were tested for CCK immunoreactivity (Fligure 4 panel A)?

Page 14. The authors should make clear in the text that these ifenprodil experiments in interneurons refer to wildtype recordings. Did the authors attempt to repeat these expts in the GluN2A KO rat slices?

Page 16 and figure 6 panel A. there is no discussion of the EPSC properties in the presence absence of NAB in the text or in the figure despite panel A showing the NAB block in the 2A null rat. Can the authors include this dataset?

Minor points

Line 277 page 9. “We observed similar NMDA/AMPA ratios of ........” this is an awkward sentence and should be altered as its meaning is unclear

Page 12 line 348 CGE should be the caudal ganglionic eminence and not the central

Page 12 line 349 “contain high levels of NMDA receptors” not NMDA

Page 12 line 354 perforant not performant

## Author Response

1

eN-NWR-0552-20R1: Response to Reviewers:

We have revised our manuscript to take account of the reviewers’ suggestions. We have

performed a number of additional experiments and completed a re-evaluation of our

statistical analysis along the lines suggested to include estimation statistics. As a

consequence of the additional work and analysis we have added an additional author to our

manuscript given the amount and nature of the work that was contributed by this individual.

REVIEWER´S COMMENTS

Reviewer #1

In this manuscript titled “Contribution of N-methyl D-aspartate receptors to synaptic function

in rat hippocampal interneurons", the authors present data that suggest the GluN2A subunit

of the NMDA receptor makes unequal contributions to synaptic transmission across various

types of interneurons in the CA1 subfield of the rat hippocampus. Using a CRISPR-Cas9

knockout rat model, the researchers delete the Grin2a gene and use the EPSC time course

as a readout of GluN2A’s influence on excitatory tone onto PV-, CCK-, and SST-expressing

interneurons. Moreover, the authors conclude that each of these interneuron subtypes

utilizes GluN2B-containing NMDA receptors for synaptic transmission via ifenprodil inhibition

of the evoked EPSC and that the GluN2D subunit expression may have important

implications for LTP onto CCK-expressing interneurons.

Overall, the rationale for this work is sound, and the information gained from this study may

help future drug development. Interneurons and the inhibitory network can be dysfunctional

in a host of neurological conditions and thus their modulation may prove therapeutically

useful. However, there are several concerns that should be addressed before publication.

We thank the reviewer for their positive appraisal of our manuscript and their appreciation of

how this work may contribute to a greater understanding of interneuron and brain function

more generally.

Major concerns:

1. There does not appear to be any justification for the number of replicates completed in the

manuscript, ranging from as little as 4 animals (5 cells) up to 24 animals (52 cells). It almost

appears as if the authors arbitrarily chose the number of replicates needed for experiment.

Without more explanation, it is not possible to ascertain why certain measures, such as the

NMDA receptor-mediated EPSC decay time for CCK-expressing interneurons required 24

animals (52 cells) while an analogous experiment, the tau decay for pyramidal cells, required

far fewer. There also is no mention of power analysis to substantiate selection of number of

observations.

We appreciate the reviewers concern regarding the number of animals recorded per cell

type for these experiments. We estimated that a minimum of 7-10 rats per group would be

sufficient to detect an effect size of 20%, assuming 15% SD and an anticipated power of

80% (testing null-hypothesis at 95% probability). Based on our experience with collecting

identified inhibitory interneurons, particularly in the absence of a interneuron reporter and

using a Cs-gluconate internal solution (which precludes identification based on action

potential discharge properties), the required number or rats to fulfil groups, such as PV of

SSt INs would be in fact much higher - following successful identification with

immunohistochemistry. However, to try to balance recordings such that all interneuron

subtypes were sampled from the greatest biologically diverse group, CCK INs were sampled

from the same animals, which were typically more reliably identified. That being said, in our

initial submission, some experimental groups in our main datasets were still underpowered.

To address the reviewers concern, we have performed a final experimental cohort to satisfy 2

the projected sample size for all groups in Figures 1-4 and have added a comment to the

statistics section to elaborate on power analysis (lines 212-221).3

2. The authors report that EPSCs recorded from WT pyramidal cells at 30 degrees C had a

weighted tau of 139 ms and that GluN2A-KO mice had a prolonged decay time (211 ms).

These data agree with previously published data. However, the weighted tau decay times for

WT PV-expressing interneurons is reported as 73 ms and SST-expressing interneurons as

59 ms. These decay values do not match previously published data, nor do they align with

the idea that GluN2D-containing NMDA receptors have been reported on both of these

interneuron subtypes. For example, Cornford et. al. 2019 (PMID: 31657720) report NMDA

receptor-mediated EPSCs, evoked via Schaffer collateral stimulation at 30 degrees C, to

have an average weighted tau decay time of roughly 150 ms. In this manuscript, the authors

report three PV-expressing interneurons to have a weighted tau decay time of less than 50

ms, and we were unable to find any report of similar values in the literature. These concerns

are also shared for SST-expressing interneurons. The authors need to compare their results

to those in the literature in order to provide both context for their results and to ensure that

the recordings/analyses are not subject to technical errors. For example, some of their

measured NMDA/AMPA ratios are near zero, raising a question as to whether the briefest

taus were subject to artifacts and noise, possibly as a result of small amplitude. Could the

authors reanalyze the deactivation data with strict cut off for response amplitude to ensure

the results were not influenced by noise or baseline drift for the smallest responses?

We have performed a careful survey of our data and indeed the reviewer is correct, the

responses with the most rapid kinetics arose from the smallest NMDAR EPSCs. To address

this issue, we imposed a threshold for kinetic measurement of NMDAR EPSCs of 20 pA.

Such responses are now only included in the measurement on basal NMDAR/AMPAR

amplitude relationships and not for kinetic analysis. A comment has been added to the

methods section to clarify this (lines 159-161).

3. The data in the time course plot in Figure 5B does not seem consistent with the bar graph

in Figure 5C. The CCK and SST plots are on top of each other in the time course plot

(Figure 5B) but show a 20% difference in the bar graph representation (Figure 5C). The time

course plot suggests there are two groups, PC/PV and CCK/SST, with clear and large

differences, but the biggest difference in the bar graph appears to only differentiate SST and CCK-expressing interneurons.

This discrepancy is due largely to not all CCK INs being included in the time-course plot. For

a number of cells, the time-course of ifenprodil wash-in was not recorded (n=5 cells), but

rather the NMDAR EPSC recorded at the same 8-10 minute period after bath application.

We have included the number of cells in the legend on the plots in Figure 5B and confirmed

that this wash-in was in a subset of cells in the figure legend.

4. A baseline period of only five minutes may be too short for a plasticity experiment, with

most studies reporting 15-20 minutes. Figure 6C almost looks as if the response amplitude is

trending upward prior to the LTP stimulus, raising a question of whether the recording was

stable before LTP induction.

The ability to induce LTP following a 100 Hz tetanus must be induced within 10-15 minutes

of breakthrough into whole-cell configuration (Kato et al., (1993) Neuroscience; Lamsa et al.,

(2005) Nature Neuroscience). Furthermore, it has been shown recently that CCK INs do

indeed possess a cytosolic calcium binding protein (NECAB1/2), which buffers intracellular

calcium (Miczán et al., (2021) Cerebral Cortex); further highlighting the importance of rapidly

inducing LTP in the whole-cell configuration.

Specifically in our experiments, establishing whole-cell recording conditions, correct bridge

balancing and wash-in of the internal solution, followed by determination of a monosynaptic 4

EPSP amplitude, took approximately 4-5 minutes. This allows us between 5-10 minutes

baseline. To ensure consistency between recordings, we aimed for 5 minute control baseline

to overcome any variability in the earlier set-up and to avoid recording for longer than 10

minutes of baseline. Cells which failed to produce a relatively stable response in this 5

minutes of EPSP baseline were excluded, based on a 10% change over baseline (lines 170-

171). We have re-analysed the recordings and found 1 cell had produced a spontaneous

action potential during the baseline of the WT data in Figure 6C, explaining this upward

tendency.

5. Figure 6A suggests that NAB-14 is less effective in blocking NMDAR responses in the 2A null mice but there appears to be no quantification of NAB-14 effects on EPSCs from 2A-null

mice.

The reviewer was quite correct in their observation. We had only recorded a minimal number

of 2A-null CCK INs in the presence of NAB-14. We have now expanded this and can

quantitatively compare GluN2A-null control vs. NAB-14 (see new panel in Figure 6C) and

associated text (line 484-497).

Minor concerns:

1. The introduction and layout of the manuscript do not provide a clear explanation of the

importance and design of the LTP experiments for only one interneuron subtype.

We have included text (lines 83-88; 519-524) to address this point.

2. The discussion does not really place the results in the context of what is known, and also

misses an opportunity to identify any weaknesses or caveats in the manuscript. For

example, the lack of GluN2A’s participation in excitatory transmission onto CCK-expressing

interneurons is not really discussed. There is also no discussion of the temporal timing of

GluN2A expression, a topic relevant for development. The authors should explicitly comment

that the study only probes feedforward inhibition, neglecting synaptic connections utilized in

feedback inhibition. The authors should also ensure they clearly articulate for those not in

the field they have focused on only a couple interneuron types, and ensure they do not leave

a reader with the impression that there only three main neurochemical markers for

interneurons in the CA1 subfield.

We have addressed these points in the discussion, as and where relevant (lines 576-582).

We should note that we previously clearly stated that we specifically examined feed-back

interneurons (SSt INs) in this study (line 141-142, 328-332), as the predominant input they

receive arises from local CA1 PyrCs.

3. 10 M ifenprodil is not the optimal concentration from which to draw conclusions about

GluN2B involvement. 3 M would have been better, since at 10 M there can be some nonselective block of GluN2A by ifenprodil. This should be raised as a caveat.

We have now included data showing that the ifenprodil block is larger in most IN subtypes in

the GluN2A-null rat, consistent with a selective effect. However, we cannot rule out a

potential non-specific action at native receptors in the wild-type rat. We have added a

comment regarding this possible caveat to the discussion (lines 576-582).

4. The lack of a change in the time course of NMDA receptor-mediated EPSCs following

NAB-14 treatment could occur if most receptors at the synapse were triheteromeric 5

receptors that contained either GluN2A/GluN2D or GluN2B/GluN2D, with no GluN2D

diheteromeric receptors. In this situation, GluN2A or GluN2B could dominate the

deactivation, and NAB-14 would simply reduce the number of active receptors. One would

only expect a change in the time course if removal of GluN2D-containing receptors revealed

another NAB-14-resistant population of NMDA receptors with a different response time

course.

To address this point we re-analysed all the NMDAR EPSC data in the presence of NAB-14.

In a number of cells, the decay time of the NMDAR EPSC was not appropriately fit due to

the smaller amplitude EPSC in the presence of NAB-14. Reanalysis revealed that the

EPSCs were indeed faster in the presence of the GluN2C/D negative allosteric modulator

both in WT and in the GluN2A-null rats (lines 484-497).

5. The authors should consider the possibility that the lack of LTP in NAB-14 is due only to

reduced NMDA activity overall and not something specific about the presence of GluN2D,

and thus perhaps a more appropriate conclusion would be that GluN2D “contributes” to LTP

and not that GluN2D “is required” for LTP.

We have toned down this line of reasoning in several places throughout the manuscript.

6. The manuscript mentions that series resistance was monitored during recording of evoked

EPSCs and cells were excluded if series resistance changed by more than 20%. However,

no further details are provided. It would be nice to see exactly how this was done and what

series resistance ranges were.

We have included detail for how series resistance was monitored. We have also now

included the range of measured series resistances of all recordings.

7. The pulse stimulation duration is not mentioned.

This was an oversight and has been added to the methods.

8. An explanation of the intensity of the voltage used to stimulate the slice is lacking and it is

unclear how the stimulus intensity was selected. Was an input-output curve generated prior

to initiation of the experiment?

We have clarified this point (line 144) and added the average stimulus intensity (line 142).

We aimed for a stimulus intensity that produced a pure monosynaptic EPSC (CA3 was cut in

all NMDAR-EPSC recordings). In some cells this was achieved at ∼200 pA, in other cells

only a lower amplitude EPSC was possible due to disynaptic contamination. This is a

consequence of recording synaptic inputs from PV and CCK INs, which both possess feed-forward and feed-back inputs within the local circuit (see Pawelzik et al., (2002) J. Comp.

Neurology). In all cells an input-output curve was minimally assessed prior to baseline

recording, however this was not consistently recorded.

9. The immunohistochemical staining of the sample images provided for SST- and CCK expressing interneurons is weak, with barely discernable somas. Perhaps these slices could

benefit from less fixation or antigen retrieval. 6

The immunolabelling for SSt and (pre-pro-) CCK is based on neuropeptide expression at the

endoplasmic reticulum. This pattern of expression does not lead to pronounced labelling of

local structures such as axon terminals or dendrites, as for PV. We have provided indicators

(asterisks) to the soma centre for these images which we hope clarify this.

10. There is no mention of a correction for family-wise error, although two measures were

statistically compared from the same recording (AMPAR/NMDAR amplitude ratio, decay

time).

As we have now included an amplitude threshold for recordings to validate kinetic property

measurement, to address the reviewers earlier comment (Major point #3) meaning that

matched observations are not present for all cells. To compare the effects of different

parameters, we have now included estimation statistics for effects size to confirm where

effects are meaningful, compared to those arising from multiple-comparisons.

11. The authors only use male rats and should provide an explanation of why females were

not included.

We chose male rats for the given the age range of 4-6 weeks, to avoid confounds arising

from the oestrus cycle, with sex hormones known to alter NMDAR signalling (Paolo 2000).

12. The criteria for termination of the experiment was described as baseline responses being

unstable but the criteria for what is stable or not is not defined.

The criteria for a stable baseline was a line fit to the baseline period, any deviation above

10% were not included in this analysis. A note has been added to the methods.

Reviewer #2

This is a very nice and straightforward examining the role of NMDAR subtypes on identified

inhibitory interneurons that takes advantage of the recently reported GluN2A null rat. The

authors demonstrate a differential role for GluN containing NMDARs and highlight that PV

and SST interneurons differ in their NMDAR composition from putative CCK-containing

interneurons in the CA1 subfield of the rat hippocampus. The data are clear and convincing

and the conclusions are warranted and supported by the data. I have only a few minor

comments that I hope the authors will find useful in revising their manuscript.

The data from both PV and SST-containing interneurons shows that a lack of GluN2A yields

NMDAR meditated currents of similar amplitude to wildtype but with longer apparent decay

kinetics as would be expected with the elimination of GluN2A. The lack of change in both the

amplitude of the NMDAR mediated current and the AMPA/NMDAR ratio is surprising, and

suggests that receptors containing GluN2A are certainly involved in this EPSC but other

subunits remain and/or compensate for the lack of GluN2A. In their summing up of this data

set (eg line 323 page 11) the authors should conclude that GluN2A likely contributes to the

observed NMDARs EPSCs but it certainly doesn’t “mediate” the synaptic current since there

appears to be sufficiently functional NMDARs in its absence. Semantics maybe, but an

important clarification nonetheless.

We have now toned down this conclusion. 7

Page 12. How certain can the authors be that all of the radiatum cells from which recordings

were made were in fact CCK-positive cells. There are many cell types that reside in the st.

radiatum in addition to the CCK-cell population. How many recovered cells were tested for

CCK immunoreactivity (Fligure 4 panel A)?

All cells included for analysis of the different cell types were confirmed by

immunohistochemistry. As such all cells included were CCK positive. We have added a line

to the results for CCK INs - but also for PV and SSt INs too.

Page 14. The authors should make clear in the text that these ifenprodil experiments in

interneurons refer to wildtype recordings. Did the authors attempt to repeat these expts in

the GluN2A KO rat slices?

We have now included new experiments that identify the ifenprodil effect in GluN2A-null rats

(Figure 5C and 5D). We found that CA1 PyrC showed a greater effect of ifenprodil in the 2A null rats, and PV and SSt INs tended to have greater block in the 2A-null line, albeit less

convincingly from estimation statistics. CCK INs showed a great overlap of their CI with WT,

consistent with no difference between genotypes and arguing that 2A contributes little to

these INs.

Page 16 and figure 6 panel A. there is no discussion of the EPSC properties in the presence

absence of NAB in the text or in the figure despite panel A showing the NAB block in the 2A

null rat. Can the authors include this dataset?

We have now included this omission in Figure 6C, as well as in text changes describing the

effect of NAB-14 on NMDAR EPSC decay time (see lines 484 - 497)

Minor points

Line 277 page 9. “We observed similar NMDA/AMPA ratios of ........” this is an awkward

sentence and should be altered as its meaning is unclear

Page 12 line 348 CGE should be the caudal ganglionic eminence and not the central

Page 12 line 349 “contain high levels of NMDA receptors” not NMDA

Page 12 line 354 perforant not performant

We have addressed all these minor issues.
